# Vitamin D–VDR Signaling Across Thyroid Disorders: A Systematic Review of Molecular Mechanisms, Autoimmunity, Genetic Susceptibility, Experimental Evidence and Therapeutic Translation

**DOI:** 10.3390/biom16101532

**Published:** 2026-10-20

**Authors:** Shakta Mani Satyam, Murk Jai Pal Paryani, Ilmia Shameer, Shams Al-dawoodi, Ebrahim Safaii, Rashmi Kumari, Abdul Rehman, Hussein Saleh, Prakashchandra Shetty, Sainath Prabhakar, Bhoomendra Bhongade, Laxminarayana Kurady Bairy, Afnan Afroz, Manasvi Udaya Kumar, Karthik Nair, Anuradha Asokan Nair

**Affiliations:** 1 Department of Pharmacology, RAK College of Medical Sciences, RAK Medical and Health Sciences University, Ras Al Khaimah 11172, United Arab Emirates; 2 A. J. Hospital and Research Centre, A. J. Institute of Hospital Management, Mangalore 575004, India; 3 Department of Pathological Sciences, College of Medicine, Ajman University, Ajman 346, United Arab Emirates; 4 Faculty of Medicine, Manipal University College Malaysia, Melaka 75150, Malaysia; 5 Manipal College of Health Professions, Manipal Academy of Higher Education, Manipal 576104, India; 6 RAK College of Pharmacy, RAK Medical and Health Sciences University, Ras Al Khaimah 11172, United Arab Emirates; 7 Department of Pharmacology, Alva’s Institute of Medical Science and Research Centre, Moodbidri 574227, India

**Keywords:** vitamin D, vitamin D receptor, VDR signaling, thyroid disorders, autoimmune thyroid disease, Hashimoto’s thyroiditis, Graves’ disease, thyroid autoimmunity, VDR polymorphisms, precision medicine

## Abstract

Vitamin D–Vitamin D receptor (VDR) signaling extends beyond calcium-phosphate homeostasis and regulates immune tolerance, inflammation, cellular differentiation, oxidative stress, and endocrine physiology. This systematic review evaluated the relationship between vitamin D–VDR signaling and thyroid disorders, including hypothyroidism, hyperthyroidism, Hashimoto’s thyroiditis, Graves’ disease, thyroid autoimmunity, genetic susceptibility, experimental mechanisms, and therapeutic implications. This study was conducted and reported in accordance with the PRISMA 2020 statement. PubMed/MEDLINE and Scopus were searched for studies published from January 2005 through June 2026, supplemented by backward and forward citation screening of relevant studies and reviews. Eligible evidence included human observational and interventional studies, genetic investigations, and relevant animal and in vitro studies addressing vitamin D status, vitamin D exposure or supplementation, VDR variation, vitamin D metabolism, or vitamin D–VDR mechanisms in relation to thyroid function, thyroid disease, thyroid autoantibodies, or related biological outcomes. A total of 13,623 records were identified, of which 11,730 unique records underwent title and abstract screening, 449 reports were assessed at the full-text stage, and 118 primary studies were included in the final synthesis. Because of substantial heterogeneity in populations, study designs, vitamin D exposures, outcomes, genetic backgrounds, and experimental models, findings were synthesized narratively rather than by quantitative meta-analysis. Lower circulating 25-hydroxyvitamin D [25(OH)D] concentrations were frequently associated with thyroid disease, particularly autoimmune phenotypes, and increased thyroid peroxidase and thyroglobulin antibodies in Hashimoto’s thyroiditis. Interventional evidence suggests that vitamin D supplementation may reduce thyroid peroxidase antibody (TPOAb) and thyroglobulin antibody (TgAb) concentrations in some patients with autoimmune thyroid disease. However, these laboratory changes represent immunological surrogate outcomes and should not be interpreted as evidence of restored thyroid function, prevention of progression to overt hypothyroidism, reduced dependence on thyroid hormone replacement, or sustained clinical remission. Randomized evidence does not establish a general preventive effect of vitamin D supplementation on incident hypothyroidism. Associations between VDR polymorphisms, including FokI/rs2228570, rs1544410, rs731236, and rs7975232, and thyroid autoimmunity were inconsistent across populations. Experimental evidence implicates immune regulation, Th17/Treg balance, inflammatory signaling, vitamin D metabolism, and calcium-phosphate homeostasis. The evidence was limited by substantial clinical, methodological, and biological heterogeneity, frequent reliance on observational studies, population-specific genetic findings, and differences between experimental models and human thyroid physiology. Overall, vitamin D–VDR signaling represents a biologically plausible modifier of thyroid disease, but current evidence does not establish universal causality or therapeutic efficacy.

## 1. Introduction

It is increasingly recognized that thyroid disease is not a single clinical entity but a spectrum of thyroid dysfunction ranging from subclinical to overt hypothyroidism and hyperthyroidism. Autoimmune thyroid diseases, including Hashimoto’s thyroiditis (HT) and Graves’ disease, are the most common underlying etiologies of both hypothyroid and hyperthyroid diseases in iodine-sufficient populations. However, despite sharing an autoimmune basis, the two diseases represent distinct clinical entities with distinct target self-antigens, immunopathogenesis, clinical manifestations and treatment approaches. Importantly, both forms of autoimmune thyroid disease are influenced by a combination of genetic and environmental determinants that interact with endocrine and immune systems to determine susceptibility and disease outcome. Therefore, when considering the relationship between vitamin D and thyroid disease, it is essential to consider the spectrum of both thyroid dysfunction and its underlying causes rather than restricting consideration to autoimmune thyroiditis. The evidence base provided herein encompasses a broad spectrum of both thyroid dysfunction and its underlying causes.

The traditional concept of vitamin D as a nutritional component for the regulation of calcium and phosphate for skeletal health is incomplete [1]. 1,25-dihydroxyvitamin D [1,25(OH)_2_D] is the biologically active form of vitamin D that binds to the vitamin D Receptor (VDR), a nuclear receptor found in high density in immune cells and in many endocrine cells including thyroid follicular cells. Activation of VDR by 1,25(OH)_2_D promotes heterodimerization with retinoid X receptor (RXR), enabling the complex to interact with vitamin D response elements (VDREs) in target regulatory regions and modulate gene transcription involved in immune cell differentiation, inflammation, cell cycle progression and general tissue homeostasis in a wide array of cells including thyroid follicular cells and their associated immune cells [2]. Evidence from thyroid-specific experimental studies has also examined the expression of VDR and vitamin D-metabolizing enzymes in thyroid tissue, including normal thyroid tissue and thyroid neoplasms [3,4,5]. These primary investigations provide tissue-level evidence relevant to the biological plausibility of vitamin D–VDR signaling in the thyroid, whereas broader reviews provide context for its potential endocrine and immunological functions. These findings provide a biological rationale for investigating vitamin D as a micronutrient with hormone-like actions that may influence endocrine and immune regulation, in addition to its established role in calcium-phosphate homeostasis and skeletal health.

In addition to its well-known effects on calcium and phosphorus metabolism, vitamin D can also exert effects on thyroidal tissue [6]. The expression of the VDR in thyroid follicular cells, as well as in lymphocytes, dendritic cells and macrophages that infiltrate the thyroid gland in autoimmune disease, has been described [7]. Moreover, the presence of the VDR in C-cells, and the regulation of C-cell gene expression by vitamin D3 in both normal and neoplastic thyrocytes, provides additional mechanisms whereby vitamin D may influence thyroidal development and function. The expression of the VDR in thyroid cells has recently been studied in more detail in a series of experiments performed using VDR deficient mice. Although selected C-cell-associated functions were affected, thyroid development and overall thyroid function were largely preserved in the VDR-deficient animals studied [3]. More broadly, preclinical studies have demonstrated that modulation of oxidative stress and cellular defense pathways can influence tissue injury and metabolic homeostasis, providing additional support for investigating interactions between endocrine, immune, and cellular stress pathways [8,9]. Evidence from related preclinical and translational research also highlights the potential relevance of immunometabolic regulation and cellular stress responses to disease mechanisms and therapeutic strategies [10,11,12].

Vitamin D has been associated with various conditions of thyroid disease. In studies conducted on patients with autoimmune thyroid disease, levels of 25(OH)D have been found to be low. Also, inverse correlation between vitamin D levels and levels of thyroid autoantibodies has been found in several studies. There is a growing body of evidence from observational studies linking vitamin D with different forms of thyroid disease. The relationship between vitamin D status and thyroid disease has been investigated in observational studies and systematic reviews; however, the interpretation of circulating 25-hydroxyvitamin D [25(OH)D] concentrations require careful consideration of analytical and biological heterogeneity [13,14,15,16]. Although serum or plasma 25(OH)D is the principal biomarker used to assess vitamin D status, results obtained using different analytical methods including immunoassays and liquid chromatography–tandem mass spectrometry may not be fully interchangeable because of differences in assay calibration, specificity, cross-reactivity, and performance across concentration ranges [17,18,19]. Consequently, apparent differences between study populations may partly reflect methodological variation rather than true differences in vitamin D status. Comparability is further limited using different operational thresholds for vitamin D deficiency, insufficiency, and sufficiency. A concentration classified as deficient in one study may be categorized differently in another, depending on the guideline, population, assay, and clinical outcome being considered. Such differences can influence reported deficiency prevalence, subgroup classification, and estimates of associations with thyroid autoantibodies or thyroid function. Therefore, the direction and magnitude of findings should be interpreted alongside the analytical method, units, reference thresholds, and baseline vitamin D status reported in each study.

Circulating 25(OH)D is also influenced by several biological and environmental factors, including season and ultraviolet-B exposure, dietary intake and supplementation, age, adiposity, physical activity, skin pigmentation, renal and hepatic function, inflammatory status, and concomitant medication use [20]. Obesity may affect the distribution and circulating concentration of this fat-soluble metabolite, whereas supplementation can alter concentrations according to dose, adherence, duration, baseline status, and the interval between administration and blood sampling [21,22]. Thyroid dysfunction may itself influence physical activity, body composition, skeletal turnover, and vitamin D metabolism, creating the possibility of reverse causation [23,24]. Accordingly, differences in 25(OH)D concentrations across studies cannot be attributed exclusively to thyroid disease or interpreted as evidence of a causal relationship. Where reported, these factors should be considered when comparing populations and interpreting associations. The heterogeneity in assay methods, classification thresholds, study populations, and potential confounders also limits the extent to which the findings can be quantitatively compared across the included studies.

The fact that the autoimmune thyroid disease forms the strongest mechanistic link between VDR, and thyroid disease is evident from the fact that the VDR-activated vitamin D3 affects dendritic cells and antigen-presenting cells as well as T- and B-cells [25]. The Vitamin D3-activated VDR is able to promote the activity of T-regulatory cells while it down-regulates the T helper 1 (Th1) as well as Th17-type cytokine profiles and the corresponding effector cells that cause thyroid follicular-cell injury through Interferon-gamma (IFN-γ), Interleukin-17 (IL-17) and Tumor necrosis factor-alpha (TNF-α), a pathway that can be affected by vitamin D3 [26,27,28]. The mere presence of VDR in the thyroid follicular cells, as well as in lymphocytes, in dendritic cells and in macrophages, provides a biological rationale for the endocrine-immune interaction studied here in the context of thyroid disease. However, VDR activity in VDR-expressing cells in general, and in the thyroid in particular, is not sufficient to explain the phenomenon of autoimmune thyroid disease.

VDR polymorphisms have been associated with HT in several studies. Wang et al. found a significant association between FokI polymorphism and the presence of HT. In contrast, no association was found for TaqI, ApaI or BsmI polymorphisms [29]. Subsequent meta-analyses including more studies, by Gao and Yu [30] and Pakosiński et al. [31], identified associations between different polymorphisms (rs731236, rs1544410, rs2228570, and rs7975232) and autoimmune thyroid diseases, including HT. However, the studies reported a considerable variability in terms of studied phenotype and studied populations.

The relationship between circulating 25(OH)D concentrations and thyroid dysfunction may be bidirectional and is potentially influenced by shared determinants. Low vitamin D status may precede thyroid disease in some individuals, but it may also arise after disease onset through changes in physical activity, body composition, dietary intake, inflammation, skeletal turnover, or vitamin D metabolism. Obesity, age, season, comorbid metabolic disease, renal or hepatic dysfunction, medication exposure, and socioeconomic or lifestyle factors may additionally influence both vitamin D status and thyroid outcomes. Consequently, the inverse association between 25(OH)D concentrations and thyroid autoantibodies reported in some populations cannot, on its own, establish that vitamin D deficiency initiates or aggravates autoimmune thyroid disease [32,33,34]. Similarly, a correlation between vitamin D status and thyroid function does not establish that supplementation will improve thyroid function [6,35,36]. Longitudinal studies with appropriate confounder adjustment and randomized intervention studies are required to distinguish potential causal effects from reverse causation and residual confounding. These studies, however, were not designed to test for disease prevention and thus cannot be used as evidence that vitamin D supplementation can prevent thyroid disease. Randomized evidence is particularly important for distinguishing observational associations from causal effects. However, whether vitamin D supplementation influences thyroid outcomes may depend on the population studied, baseline vitamin D status, intervention regimen, and clinical endpoint.

The relationship between vitamin D–VDR signaling and thyroid autoimmunity depends on the underlying thyroid phenotype [37,38]. Hashimoto’s thyroiditis is characterized by immune-mediated injury to thyroid follicular cells, commonly accompanied by TPOAb and/or TgAb positivity, and may progress to hypothyroidism as functional thyroid tissue is lost [39]. Graves’ disease is characterized by TSH-receptor-directed autoimmunity, in which stimulating antibodies activate the TSH receptor and contribute to hyperthyroidism. In these two autoimmune conditions, vitamin D–VDR signaling is of interest because of its potential effects on antigen presentation, T-cell differentiation, regulatory immune pathways, and antibody production. By contrast, hypothyroidism following thyroidectomy or radioactive iodine treatment is primarily related to loss of functioning thyroid tissue and should not automatically be interpreted as ongoing autoimmune thyroid destruction. Similarly, hyperthyroidism is a physiological phenotype rather than a single etiology and may arise from autoimmune or non-autoimmune causes. Consequently, studies should distinguish thyroid functional status from the underlying disease mechanism when evaluating associations with vitamin D status or VDR signaling.

In a meta-analysis of 10 clinical studies including 577 patients with autoimmune thyroid disease, the levels of Thyroid peroxidase antibodies (TPOAb) and Thyroglobulin antibodies (TgAb) decreased significantly after vitamin D supplementation [40]. Some of the studies have reported reductions in TPOAb and/or TgAb concentrations following vitamin D supplementation in patients with autoimmune thyroid disease [40,41]. Nevertheless, the magnitude and consistency of these changes may vary according to baseline vitamin D status, supplementation regimen, treatment duration, population characteristics, and study design. Autoantibody concentrations are immunological biomarkers rather than direct measures of thyroid functional recovery. Consequently, reductions in TPOAb or TgAb should be interpreted as evidence of a possible immunomodulatory effect and not, in isolation, as evidence of clinical improvement. However, it must be emphasized that decreases in thyroid autoantibodies have not yet been shown to translate into a cure of hypothyroidism (either permanent or temporary) in humans. In other words, while vitamin D may have a therapeutic effect on the immune system of patients with autoimmune thyroid diseases, it is likely to have little or no therapeutic effect on the endocrine tissue itself. Vitamin D plays a clinically important role in the course of thyroid diseases during the preoperative and postoperative periods of thyroid surgery, especially because of the possibility of a transient parathyroid dysfunction with an interference in calcium metabolism. In this specific clinical setting, the role of vitamin D in calcium metabolism has been reviewed by Canali et al. [42]. Therefore, the preoperative and postoperative periods of thyroid surgery represent a distinct and clinically relevant clinical setting within the broad spectrum of relationships between vitamin D and thyroid diseases.

The rationale for the present systematic review therefore arises from the fragmentation of existing evidence. Studies have investigated vitamin D deficiency, autoimmune thyroid disease, thyroid hormone concentrations, VDR polymorphisms, calcium homeostasis, metabolic disease and experimental mechanisms separately. A comprehensive synthesis that distinguishes hypothyroidism from hyperthyroidism, and both from their autoimmune etiologies, while integrating clinical, genetic, experimental and perioperative evidence, remains necessary. The present review addresses this gap by integrating clinical, genetic, and experimental evidence within an endocrine–immune–genomic framework. The present review was therefore designed to determine whether the available evidence supports a coherent biological model of vitamin D–VDR involvement across thyroid disease and to identify which aspects of this pathway have sufficient evidence to justify further preclinical and clinical development.

## 2. Methodology

The present systematic review was conducted and reported in accordance with the Preferred Reporting Items for Systematic Reviews and Meta-Analyses (PRISMA) 2020 statement [43]. The review was designed to comprehensively evaluate the relationship between vitamin D–vitamin D receptor (VDR) signaling and thyroid disorders, integrating clinical, epidemiological, genetic, mechanistic, animal and in vitro evidence. The review addressed autoimmune and non-autoimmune thyroid disorders, including hypothyroidism, hyperthyroidism, Hashimoto’s thyroiditis, Graves’ disease and related thyroid phenotypes, with particular emphasis on vitamin D status, vitamin D supplementation, VDR signaling, VDR-related genetic susceptibility, thyroid autoimmunity, endocrine and metabolic outcomes, and mechanistic evidence relevant to therapeutic translation. Studies published between January 2005, and June 2026 were identified in PubMed/MEDLINE and Scopus via a systematic search using controlled vocabulary and free-text terms, with Boolean operators, truncation and database-specific syntax. The core search for vitamin D and Thyroid Diseases was: (“vitamin D”[MeSH Terms] OR “25-Hydroxyvitamin D”[MeSH Terms] OR “vitamin D” OR “25-hydroxyvitamin D” OR cholecalciferol OR calcitriol) AND (“Thyroid Diseases”[MeSH Terms] OR thyroid OR hypothyroidism OR hyperthyroidism OR Hashimoto* OR Graves* OR “Hashimoto thyroiditis” OR “Graves’ disease”). The VDR/genetic search was: (“Vitamin D Receptor”[MeSH Terms] OR “vitamin D receptor” OR VDR OR polymorphism* OR SNP* OR rs2228570 OR rs1544410 OR rs731236) AND (thyroid OR hypothyroidism OR hyperthyroidism OR Hashimoto* OR Graves*). The searches were extended by combining vitamin D/VDR terms with thyroid terms and by adding thyroid autoantibodies and vitamin D metabolic pathway terms (TPOAb, TgAb, TRAb, CYP27B1, CYP24A1, calcium and phosphate metabolism, thyroidectomy and radioactive iodine ablation, pediatric thyroid disease, studies of cytokines and T-cell subsets, and metabolic parameters). Database-specific syntax was used in Scopus searches. The reference lists of included studies and of relevant systematic reviews were examined for other relevant primary studies, with backward and forward citation tracing where appropriate. Eligible evidence included human observational and interventional studies, genetic association studies, relevant animal studies and in vitro/mechanistic investigations that addressed a relevant relationship between vitamin D or VDR signaling and thyroid disease or thyroid-related biological outcomes. Studies were considered eligible when they provided information directly relevant to the review objectives, including vitamin D status or supplementation, VDR expression or signaling, VDR-related genetic variation, thyroid function, thyroid autoimmunity, thyroid autoantibodies, calcium-phosphate homeostasis in relevant thyroid settings, or mechanistic pathways linking vitamin D–VDR signaling with thyroid disease. Relevant systematic reviews and meta-analyses were considered primarily for contextual interpretation and identification of additional primary evidence. Case reports and case series involving fewer than five patients were generally excluded unless they provided particularly important mechanistic information. Studies without relevant vitamin D/VDR or thyroid-related outcomes and studies published in languages other than English were excluded.

**Figure 1:**
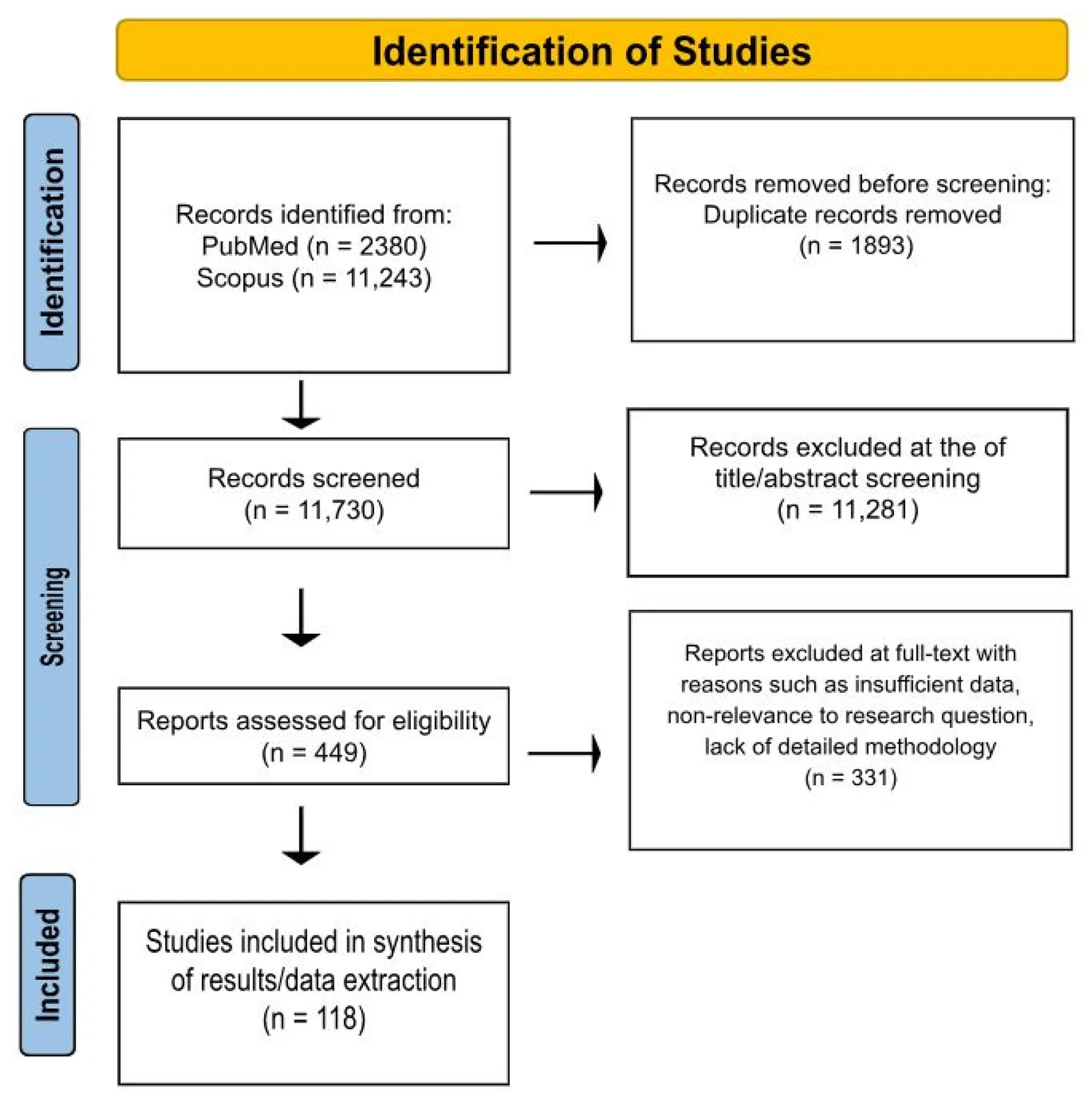
Flowchart showing the process of article selection.

Study selection was performed in multiple-reviewer stages. Following removal of duplicate records, the retrieved records were screened based on title and abstract for potential relevance to the review question. Potentially eligible reports were subsequently assessed in full text against the predefined eligibility criteria. Disagreements or uncertainty regarding eligibility were resolved through discussion among the review team and consideration of the predefined objectives and eligibility criteria. The selection process and numbers of records retained at each stage are presented in the PRISMA 2020 flow diagram (Figure 1).

Figure 1 depicts the study identification and selection process according to the PRISMA 2020 framework. The searches of PubMed/MEDLINE and Scopus identified 13,623 records published between January 2005 and June 2026. After removal of 1893 duplicate records, 11,730 unique records underwent title and abstract screening, of which 11,281 were excluded because they were not considered relevant to the review question or did not meet the predefined eligibility criteria. A total of 449 reports were subsequently assessed at the full-text stage. Of these, 331 reports were excluded because they did not meet the predefined eligibility criteria and/or were not sufficiently relevant to the review question and objectives. A total of 118 primary studies met the eligibility criteria and were included in the qualitative synthesis and data extraction.

Data were extracted from the included studies using a structured approach and were checked within the review team before synthesis. Extracted information included study design, study population or experimental model, thyroid phenotype, vitamin D exposure or intervention, vitamin D status or relevant metabolites, VDR-related characteristics, genetic variants where applicable, relevant biological or clinical outcomes, principal findings and their relevance to the review objectives. For experimental studies, information concerning the model, intervention or exposure, mechanistic pathway and principal biological outcomes was also considered. The principal outcome domains included thyroid function measures such as thyroid-stimulating hormone (TSH), free thyroxine (T4) and free triiodothyronine (T3); thyroid autoantibodies including thyroid peroxidase antibodies (TPOAb), thyroglobulin antibodies (TgAb) and thyrotropin receptor antibodies (TRAb); circulating vitamin D measures, particularly 25-hydroxyvitamin D [25(OH)D]; VDR expression and signaling; genetic variants involving VDR and related vitamin D metabolic pathways; calcium, phosphate and parathyroid hormone (PTH) parameters where relevant; and immune, inflammatory, metabolic and mechanistic outcomes relevant to vitamin D–VDR signaling and thyroid disease. Study characteristics and contextual variables, including age, sex, disease phenotype, baseline vitamin D status, intervention characteristics, duration of follow-up, experimental model and relevant genetic background, were considered where reported. No formal risk-of-bias assessment was performed in this review. Accordingly, the review does not assign formal low, moderate or high risk-of-bias judgements to individual studies. Methodological characteristics and potential limitations of the evidence were considered descriptively during interpretation, including study design, adequacy of controls, reproducibility, sample size, methodological transparency and appropriateness of the reported analyses where these features were available. For animal and in vitro studies, relevant methodological and reporting characteristics were considered when interpreting the strength and translational relevance of the findings; however, these assessments were not treated as formal risk-of-bias ratings. Because of substantial heterogeneity among the included studies with respect to populations, thyroid phenotypes, vitamin D exposure and supplementation regimens, study designs, genetic backgrounds, experimental models, outcome definitions and measurement approaches, quantitative meta-analysis was not considered appropriate for the overall evidence base. The findings were therefore synthesized narratively and organized according to thyroid disease phenotype and major evidence domains, including thyroid autoimmunity, thyroid function, genetic susceptibility, metabolic and skeletal consequences, perioperative settings, and experimental mechanisms. Findings were compared according to study design, direction and consistency of associations, biological plausibility, intervention characteristics and translational relevance. No formal subgroup meta-analysis, meta-regression or quantitative sensitivity analysis was performed. The present review did not formally assess publication bias or other reporting bias using statistical methods such as funnel plots or small-study effect tests because no quantitative meta-analysis was undertaken. Potential reporting limitations were considered qualitatively during interpretation, particularly in relation to the predominance of observational, mechanistic and experimental evidence in several domains. Similarly, no formal GRADE or other validated certainty-of-evidence framework was applied because the review incorporated heterogeneous clinical, genetic, animal and in vitro evidence that could not be readily evaluated within a single certainty framework. Accordingly, conclusions regarding the strength of evidence were based on the consistency, biological plausibility, study design and convergence of findings across evidence types rather than on a formal certainty rating. This systematic review was not prospectively registered in PROSPERO or another systematic review registry, and no formal review protocol was prepared or made publicly accessible before the review was conducted. The review was nevertheless structured around predefined eligibility criteria, systematic database searches, a prespecified review objective, multistage study selection and structured qualitative synthesis, and is reported according to the PRISMA 2020 statement. No amendments to a registered protocol were therefore applicable. No human participants or animals were directly recruited or experimentally manipulated for the purposes of this systematic review; therefore, institutional ethical approval and informed consent were not applicable.

## 3. Results

### 3.1. Study Identification, Selection and Overall Evidence Architecture

The 118 included primary studies represented a heterogeneous evidence base comprising clinical, epidemiological, metabolic, pediatric, perioperative, genetic, mechanistic and animal investigations. Autoimmune studies examined associations between vitamin D status and thyroid autoantibodies, whereas metabolic and endocrine studies evaluated relationships involving thyroid function, obesity, diabetes and related metabolic parameters. Perioperative studies addressed vitamin D, calcium and PTH in the context of thyroid surgery and radioactive iodine treatment. Pediatric studies evaluated vitamin D status in children and adolescents with thyroid dysfunction, while experimental studies investigated potential mechanisms linking vitamin D–VDR signaling with immune regulation, inflammation and thyroid-related biological processes. Randomized trials and meta-analyses were considered alongside observational and experimental evidence to assess whether clinical observations were supported by intervention data and mechanistic plausibility. Given the heterogeneity of study designs and outcomes, the evidence was integrated qualitatively rather than quantitatively. Table 1 summarizes the clinical, genetic, and tissue-level evidence supporting the role of vitamin D status and VDR signaling in thyroid disease phenotypes.

### 3.2. Characteristics of the Included Populations and Clinical Contexts

The study populations consisted of mixed ethnic groups, with most of the populations being female and of middle to old age, since autoimmune thyroid diseases are more common in females and in middle to old age. There were also considerable proportions of subjects with metabolic comorbidities, such as obesity, prediabetes and type 2 diabetes. The levels of 25(OH)D were inversely related to Body mass index (BMI), fasting glucose, Glycated hemoglobin (HbA1c) and other cardiometabolic traits [63,64,65]. One of the studies investigated weight-loss trajectories in relation to changes in 25(OH)D and TSH and found that participants who lost weight had increases in 25(OH)D and decreases in TSH, whereas those who gained weight did not [66]. However, this does not provide direct evidence for a vitamin D-TSH axis pathway since weight loss per se can affect the thyroid axis. There was considerable heterogeneity between studies for the different autoimmune traits, particularly for anti-DFS70 autoimmunity, where no significant differences in vitamin D levels were found between autoimmune disease-positive and -negative groups [67,68,69]. Pediatric populations represented a unique group in this review since growth, puberty and bone maturity as well as age-specific thyroid function references all need to be considered when studying vitamin D effects and deficiency in children and adolescents [70,71]. Perioperative populations represented a clinically relevant group with specific problems, i.e., disturbances in calcium and PTH levels and hypocalcemia postoperatively [72,73].

### 3.3. Vitamin D Status in Hypothyroidism

In the included observational studies, lower 25(OH)D concentrations were frequently reported in patients with hypothyroidism, particularly when autoimmune thyroid disease was present [55,74,75,76]. However, these findings should be interpreted as associations because the studies differed in population characteristics, thyroid disease etiology, baseline vitamin D status, and adjustment for potential confounders. The evidence does not establish that vitamin D deficiency is a necessary or sufficient cause of hypothyroidism. The evidence included in the present review relates to the specific populations of participants in the individual studies, i.e., autoimmune thyroid disease (ATD), post-thyroidectomy (PT) or Post-radioiodine (PR) hypothyroidism. The key observation from the evidence was that in populations with thyroid disease, many had low levels of vitamin D and that hypothyroid disease, particularly autoimmune in origin, was identified as one of the groups with vitamin D deficiency in the source data set. However, vitamin D deficiency may not be due to a primary deficiency of vitamin D but could be a consequence of reduced physical activity and altered body composition due to reduced thyroid hormone action, autoimmune inflammation affecting vitamin D metabolism, or even thyroid dysfunction affecting skeletal and calcium metabolism, with shared metabolic risk factors influencing both thyroid status and vitamin D status. Hypothyroidism arising after thyroidectomy or radioactive iodine treatment should be distinguished from autoimmune hypothyroidism because the principal mechanism is loss of functioning thyroid tissue rather than necessarily ongoing immune-mediated destruction. This distinction is important when interpreting studies of vitamin D status and thyroid outcomes.

Similar evidence regarding the potential use of vitamin D to prevent hypothyroidism came from randomized controlled trials (RCTs). There was one weight-loss trajectory study in which participants who achieved a weight loss of greater than 10% body weight demonstrated increases in 25(OH)D levels and decreases in TSH levels [77]. However, participants who gained weight did not show the same increases in 25(OH)D levels and decreases in TSH levels. The D-Health trial analysis evaluated the incidence of hypothyroidism among older Australian participants randomized to monthly vitamin D_3_ supplementation (60,000 IU) or placebo. The intervention did not significantly reduce incident hypothyroidism in the population studied [44]. This finding argues against a general preventive effect of the tested regimen in that population, although it does not exclude effects in other populations or among individuals with established vitamin D deficiency. However, there was a significant increase in 25(OH)D levels in the vitamin D group. Importantly, the findings from the D-Health trial need to be contrasted with the observational data. The findings from the D-Health trial suggest that high-dose vitamin D given intermittently to relatively vitamin D-replete individuals does not decrease the incidence of hypothyroidism. Therefore, currently, there is no evidence to support the use of vitamin D to prevent hypothyroidism. It is also important to differentiate between individuals with autoimmune thyroid disease and those with non-autoimmune thyroiditis (such as that occurring following thyroidectomy or radioactive iodine therapy for treatment of hyperthyroidism). The latter individuals have lost thyroid tissue through treatment for hyperthyroidism and thus, biologically speaking, it is less likely that vitamin D will have a disease-modifying effect. All future studies on hypothyroidism should categorize the participants according to the cause of their disease.

### 3.4. Vitamin D Status in Hyperthyroidism

However, hyperthyroid diseases affect bone and mineral metabolism. Hyperthyroidism increases bone resorption and alters calcium metabolism which can impact on vitamin D status and requirements [78,79]. Experimental studies have demonstrated reduced 25(OH)D concentrations during thyrotoxicosis, while observations in human hyperthyroidism suggest that increased catabolism of vitamin D metabolites may contribute to low vitamin D status in some patients [6,45,80]. These findings are important because they indicate that vitamin D deficiency in hyperthyroidism may not simply represent a pre-existing nutritional abnormality but may, at least in part, be a consequence of the hyperthyroid state itself. Such data highlight that vitamin D deficiency may be a consequence of hyperthyroid disease. There are significant data to support the impact of excess thyroid hormone on bone in hyperthyroid patients. Recent studies are now starting to explore the relationship between vitamin D status, vitamin D catabolism and functional vitamin D resistance in autoimmune hyperthyroid diseases such as Graves’ disease [46,81,82]. It is becoming clear that low vitamin D levels in these patients may be due to a variety of factors including reduced dietary intake and/or decreased availability of vitamin D due to increased body mass or reduced exposure to sunlight [14,80,83]. Other factors, such as increased inflammatory markers may also play a role and result in increased catabolism of 1,25(OH)2D [84,85]. Thus, it is possible that vitamin D deficiency could worsen some of the skeletal consequences of hyperthyroidism, but evidence yet is largely anecdotal.

On the other hand, recent observations have suggested that optimal vitamin D status is associated with decreased levels of TRAb, and a higher rate of remission in patients with Graves’ disease. Although these studies were conducted in a real-world setting, and therefore their results are solely observational, it is crucial to conduct randomized controlled trials (RCTs) to assess the role of vitamin D in the treatment of Graves’ disease as potential disease-modifying treatment [47,48,49].

### 3.5. Vitamin D in Hashimoto’s Thyroiditis

Autoimmune hypothyroidism, particularly HT (Hashimoto’s thyroiditis), represents the principal clinical context in which vitamin D status and thyroid autoantibodies have been investigated together. The strongest signal for the relationship between vitamin D status and thyroid disease was for HT and the concentration of thyroid autoantibodies (TPOAb and TgAb) [86,87]. In several observational studies, the 25(OH)D concentration was found to be lower in individuals with HT compared to healthy control subjects [41,50,86]. Importantly, several studies also found inverse associations between 25(OH)D concentrations and the concentration of both TPOAb and TgAb [51,52,53,54]. The evidence, therefore, supports a positive effect of vitamin D on autoimmune thyroiditis through several mechanisms that are potentially impacted by VDR signaling including: dendritic cell immunogenicity; the function of T cells; and the proliferation and production of immunoglobulins by B cells. In one of the few intervention studies for autoimmune thyroid disease included in this review, Luo et al. (2025) performed a meta-analysis of the effect of vitamin D supplementation on the serum concentrations of TPOAb and TgAb in individuals with autoimmune thyroid disease and found that supplementing with vitamin D resulted in decreased concentrations of both autoantibodies [40]. The evidence concerning thyroid function must be evaluated separately from that concerning autoantibody concentrations, because biochemical changes in immunological markers have not consistently been accompanied by demonstrated improvements in thyroid function or clinically meaningful disease outcomes.

It is however important to distinguish between a decrease in autoantibody production and the potential clinical effects of such a decrease. Reductions in Thyroid peroxidase (TPO) and/or Tg antibodies have been shown to be of potential benefit to patients with autoimmune thyroid disease; however, the evidence to date does not support the use of vitamin D to prevent the progression from subclinical to overt hypothyroidism, to reduce the requirement for thyroid hormone in patients with treated hypothyroidism or to normalize thyroid function in patients with autoimmune thyroid disease [55,88]. Thus, vitamin D may have some benefits for individuals with autoimmune thyroid disease through its effects upon autoantibody production, but it is not a replacement for established therapies for autoimmune thyroid disease or for thyroid hormone replacement therapy in patients with hypothyroidism.

### 3.6. Vitamin D in Graves’ Disease

The relationship between vitamin D status and Graves’ disease is less clear than for Hashimoto’s. One real-world study demonstrated that maintenance of optimal levels of 25(OH)D was associated with decreased levels of TRAb and an increased rate of remission in patients with Graves’ disease [48]. This observation suggests that VDR-mediated immune regulation could extend to antibody-mediated stimulation of the thyroid gland. However, several confounders could also explain the observed decrease in TRAb with increasing levels of 25(OH)D, including increased metabolic rate with active disease, increased skeletal turnover with severe disease, and altered vitamin D metabolism with inflammation. Rather than thinking of vitamin D as a therapeutic option for the treatment of Graves’ disease, it is more appropriate to perform longitudinal intervention studies in newly diagnosed patients with recently developed Graves’ disease. These studies should assess all appropriate endpoints, including duration of remission and relapse as well as requirement for antithyroid medications, degree of thyroid hormone suppression, degree of exophthalmos, and degree of bone turnover.

Several confounders must be considered in the association between vitamin D and Graves’ disease, e.g., in severely affected patients vitamin D levels might even decrease due to higher metabolic rate and higher bone turnover. Conversely, vitamin D deficiency could influence T-cell and B-cell regulation and thus affect production of TRAb [89,90]. Long-term intervention studies are needed in newly diagnosed patients with Graves’ disease to clarify the role of vitamin D, preferably stratified for VDR-genotypes and different categories of vitamin D status at baseline. Besides changes in TRAb levels, remission rates, frequency of relapse, degree of ophthalmopathy, need for antithyroid drugs, degree of normalization of thyroid function, and effects on bone density must be considered as clinical endpoints. Vitamin D supplementation cannot be used as an alternative to established therapies of hyperthyroidism, i.e., antithyroid drugs, radioiodine, and surgery.

### 3.7. VDR Polymorphisms and Genetic Determinants of Thyroid Disease

**Figure 2:**
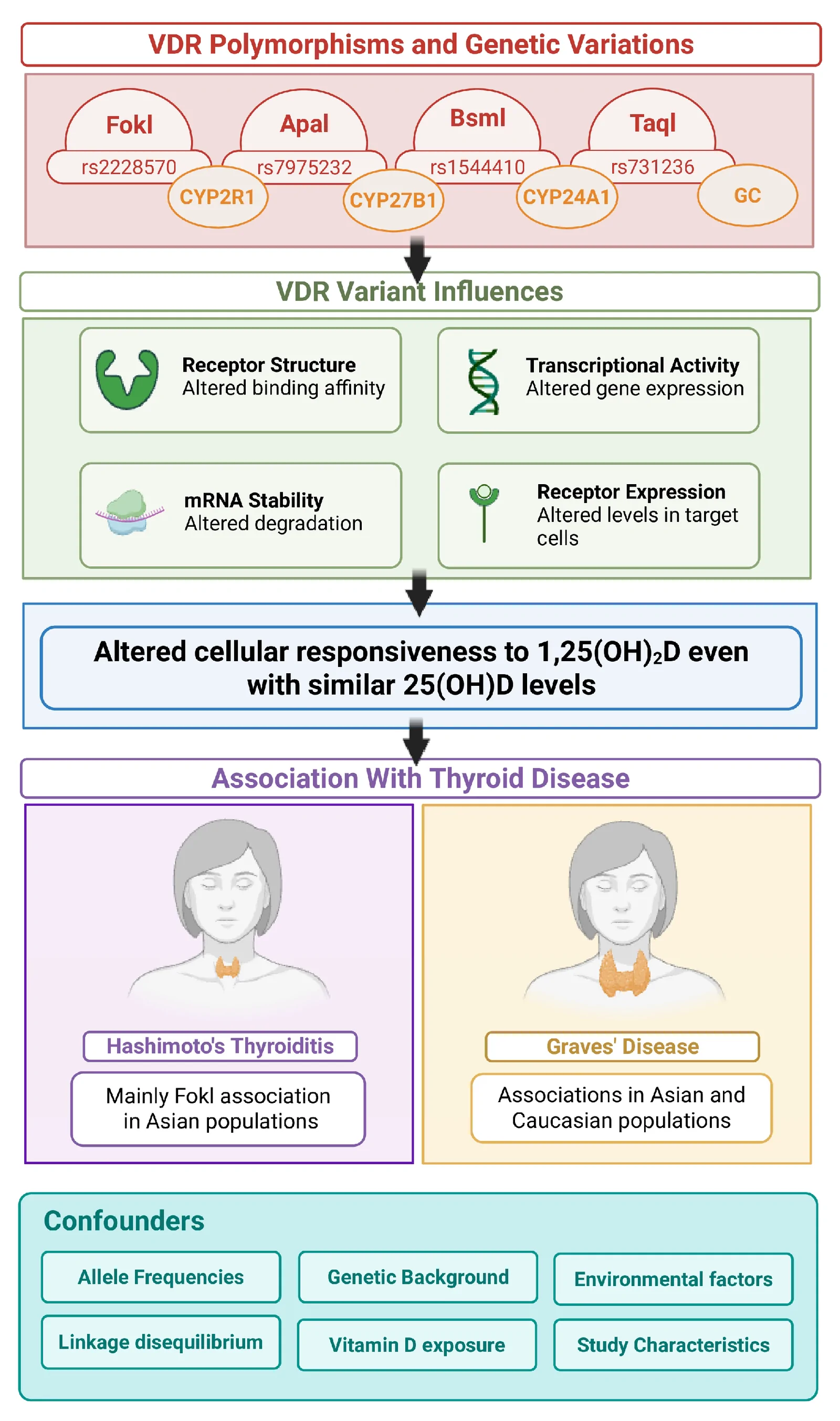
VDR polymorphisms and genetic determinants of vitamin D signaling in thyroid disease. Abbreviations: 25(OH)D, 25-hydroxyvitamin D; 1,25(OH)_2_D, 1,25-dihydroxyvitamin D; CYP2R1, cytochrome P450 family 2 subfamily R member 1; CYP24A1, cytochrome P450 family 24 subfamily A member 1; CYP27B1, cytochrome P450 family 27 subfamily B member 1; GC, vitamin D-binding protein gene; VDR, vitamin D receptor. Created in BioRender. Satyam, D. S. M. (2026) https://BioRender.com/36i69rw.

Genetic variation within the VDR pathway represents an important potential determinant of interindividual differences in vitamin D signaling and susceptibility to autoimmune thyroid disease. The VDR gene is located on chromosome 12q13.11 and contains several commonly investigated single-nucleotide polymorphisms (SNPs), among which FokI (rs2228570), BsmI (rs1544410), ApaI (rs7975232), and TaqI (rs731236) have received the greatest attention in relation to autoimmune and endocrine disorders [56,91,92,93]. The potential functional consequences of these variants and their relevance to thyroid disease are summarized in Figure 2. These variants may influence VDR-mediated biological activity through effects on receptor structure, transcriptional regulation, mRNA stability, or linkage disequilibrium with other functional variants [29,57]. Consequently, genetic variation in VDR may contribute to differences in cellular responsiveness to [1,25(OH)_2_D], even when circulating 25(OH)D concentrations are similar between individuals. This provides a biologically plausible explanation for why serum vitamin D concentration alone may not fully capture the functional activity of the vitamin D–VDR axis in thyroid disease.

Figure 2 represents commonly studied VDR polymorphisms and related vitamin D pathway genes and their potential contribution to interindividual variation in vitamin D–VDR signaling and thyroid disease susceptibility. FokI (rs2228570), ApaI (rs7975232), BsmI (rs1544410), and TaqI (rs731236) may influence VDR-related biological activity through effects on receptor structure, transcriptional activity, mRNA stability, receptor expression, or linkage disequilibrium with other functional variants. Variants in CYP2R1, CYP27B1, CYP24A1, and GC may additionally modify vitamin D transport, activation, or catabolism. These genetic differences may contribute to altered cellular responsiveness to 1,25(OH)_2_D despite similar circulating 25(OH)D concentrations. Reported associations with Hashimoto’s thyroiditis and Graves’ disease vary according to population, disease phenotype, allele frequencies, genetic background, vitamin D exposure, environmental factors, and study characteristics.

Among the four commonly studied variants, FokI (rs2228570) has attracted particular interest because it is located at the translation-initiation region of VDR and can generate receptor isoforms with different lengths and potentially different transcriptional activity. A meta-analysis by Wang et al. (2017) evaluated the association of four VDR polymorphisms (FokI, TaqI, ApaI, and BsmI) with Hashimoto’s thyroiditis [29]. The association between FokI and Hashimoto’s thyroiditis was statistically significant in the overall analysis and in the Asian subgroup, but was not statistically significant in the Caucasian subgroup, in which the association was also evaluated. In contrast, TaqI, ApaI, and BsmI were not significantly associated with Hashimoto’s thyroiditis in the overall analysis. These findings suggest that the association between VDR FokI and Hashimoto’s thyroiditis may vary across populations; however, the absence of statistical significance in the Caucasian subgroup should not be interpreted as definitive evidence of no association. The limited number of contributing studies and potential differences in statistical power and genetic background warrant cautious interpretation. These findings indicate a possible population-dependent association rather than establishing a causal role for VDR polymorphisms in the development of Hashimoto’s thyroiditis.

A broader meta-analysis by Gao and Yu that evaluated VDR rs731236, rs1544410, rs2228570, and rs7975232 in autoimmune thyroid disease further illustrates this complexity [30]. The analysis identified associations between several VDR variants and autoimmune thyroid disease, but the direction and magnitude of associations varied according to the specific polymorphism, clinical phenotype, and ethnicity. For example, rs731236 and rs2228570 were associated with altered susceptibility predominantly in HT, whereas the associations involving rs1544410 and rs7975232 differed substantially between European, Asian, and African populations. Such findings are more consistent with a population- and disease-context-dependent genetic contribution than with a single universal VDR genotype that determines autoimmune thyroid disease susceptibility.

The distinction between HT and Graves’ disease is particularly relevant because the immunopathological mechanisms underlying these disorders are not identical [94]. HT is characterized predominantly by autoreactive T- and B-cell responses directed against thyroid antigens, with progressive thyroid follicular destruction and frequent development of anti-thyroid peroxidase and anti-thyroglobulin antibodies [95,96]. Graves’ disease, in contrast, is driven principally by stimulatory autoantibodies against the thyrotropin receptor, leading to sustained thyroid stimulation and hyperthyroidism [97]. Because VDR signaling influences antigen-presenting cells, T-cell differentiation, regulatory T-cell activity, B-cell function, and cytokine production, VDR variants could theoretically modify these disease pathways differently [98]. Studies support the possibility that VDR polymorphisms may be related not only to susceptibility to autoimmune thyroid disease but also to disease phenotype or activity [31,98]. Studies evaluating patients with Graves’ disease and Hashimoto’s disease reported differences in the distribution of VDR variants between disease groups and suggested relationships between selected genotypes and Graves’ disease activity, although the relatively limited sample size and observational design require cautious interpretation [58,59].

Evidence from Graves’ disease is similarly heterogeneous. An earlier meta-analysis incorporating 1820 patients with Graves’ disease and 2066 controls reported associations of ApaI, BsmI, and FokI with susceptibility to Graves’ disease in Asian populations, whereas comparable associations were not observed in Caucasian populations [57]. When considered together with subsequent meta-analyses of autoimmune thyroid disease, these findings indicate that the relationship between VDR genotype and thyroid autoimmunity is unlikely to be adequately represented by a single pooled estimate across all ethnicities and clinical phenotypes. Differences in allele frequencies, linkage disequilibrium, sample size, diagnostic criteria, control selection, vitamin D exposure, and environmental background can all contribute to between-study heterogeneity.

The potential immunological relevance of VDR polymorphisms is biologically plausible because VDR signaling is integrated into several pathways involved in thyroid autoimmunity. Activated vitamin D can modulate antigen-presenting-cell function, influence the balance between pro-inflammatory and regulatory T-cell populations, and affect B-cell proliferation and antibody production [89,99]. Accordingly, a genetically determined alteration in VDR expression or transcriptional activity could modify the threshold at which immune tolerance is maintained. In HT, this could potentially influence the development or persistence of thyroid-directed autoimmunity, whereas in Graves’ disease it could influence the magnitude or persistence of thyrotropin receptor-directed immune responses. However, these mechanistic relationships should be regarded as biologically plausible rather than definitively established in human thyroid disease, because most available genetic studies demonstrate statistical associations rather than direct genotype-to-function relationships.

An additional consideration is that the commonly studied VDR SNPs should not necessarily be regarded as independent functional variants. BsmI, ApaI, and TaqI are located near the 3′ region of the VDR gene and may act partly through linkage disequilibrium with other functional variants rather than exerting strong independent effects themselves. Conversely, FokI is positioned at the translation initiation site and has a more direct mechanistic rationale for altering receptor function [100]. Nevertheless, even when a statistical association is observed, it does not establish that the polymorphism itself is causative. Associations may reflect linkage with another causal variant or interaction with other genes involved in vitamin D metabolism, transport, and signaling. This distinction is particularly important when interpreting candidate-gene studies conducted in relatively small case-control populations.

The genetic architecture of the vitamin D pathway also extends beyond VDR itself. Variants affecting vitamin D transport and metabolism, including genes encoding vitamin D-binding protein (GC), Cytochrome P450 family 2 subfamily R member 1 (CYP2R1), CYP27B1, and CYP24A1, could influence the availability, activation, or degradation of vitamin D and thereby modify the phenotypic consequences of VDR variation [101,102]. Indeed, studies of autoimmune thyroid disease have investigated combinations of VDR, GC, and CYP2R1 variants, suggesting that susceptibility and disease activity may reflect interactions across multiple components of the vitamin D pathway rather than the effect of a single VDR polymorphism [60,103,104]. This concept is particularly relevant to the interpretation of apparently discordant clinical findings, because an individual carrying a VDR variant may simultaneously have genetic or environmental determinants that increase vitamin D availability, alter its intracellular metabolism, or modify receptor activation.

The marked influence of ethnicity also highlights the importance of gene–environment interactions. Vitamin D status is determined not only by genotype but also by ultraviolet-B exposure, latitude, season, skin pigmentation, dietary intake, supplementation, adiposity, age, and comorbid disease [105,106]. Consequently, the same VDR variant may have different phenotypic consequences in populations with substantially different distributions of vitamin D exposure and metabolic characteristics [101,107]. The observation that associations for several VDR variants differ between Asian, European, and African populations therefore should not be interpreted simply as inconsistency; rather, it may indicate context-dependent effects arising from differences in genetic background and environmental exposure. At the same time, population stratification can produce spurious associations when cases and controls are not adequately matched for ancestry, making replication in independent and ethnically diverse cohorts essential.

These considerations have important implications for the interpretation of vitamin D supplementation studies in autoimmune thyroid disease. Although VDR genotype could theoretically influence the biological response to vitamin D, current genetic association data are insufficient to support the use of FokI, BsmI, ApaI, or TaqI as established pharmacogenomic biomarkers for selecting patients for vitamin D supplementation. An association between a VDR variant and disease susceptibility does not necessarily imply an association with treatment response. Demonstrating true pharmacogenomic relevance would require prospective intervention studies in which participants are genotyped before treatment and clinical outcomes are subsequently compared according to genotype under standardized vitamin D exposure. Relevant endpoints should include changes in TPOAb, TgAb, or TRAb concentrations, TSH and thyroid hormone concentrations, vitamin D metabolites, clinical disease activity, and, where appropriate, requirements for antithyroid or thyroid-replacement therapy.

Future studies should therefore move from conventional candidate-gene association analyses toward integrated genotype–phenotype investigations incorporating VDR variants with circulating 25(OH)D and [1,25(OH)2D] concentrations, vitamin D-binding protein, CYP2R1, CYP27B1 and CYP24A1 variants, VDR expression, downstream VDR-responsive genes, and relevant immune phenotypes. Larger multi-ethnic prospective cohorts would be particularly valuable for determining whether specific genetic backgrounds modify the relationship between vitamin D status and thyroid autoimmunity. In parallel, randomized vitamin D intervention trials incorporating prespecified genetic stratification could determine whether VDR genotype modifies biochemical or clinical response to supplementation. Such an approach would help distinguish genetic susceptibility to thyroid autoimmunity from genetic modification of vitamin D responsiveness, an important distinction that remains unresolved in the current literature.

Overall, available evidence supports a potential contribution of VDR genetic variation to autoimmune thyroid disease, but the effect appears to be heterogeneous and dependent on the specific polymorphism, thyroid phenotype, ethnicity, and broader genetic and environmental context. The current evidence therefore supports VDR polymorphisms as plausible modifiers of vitamin D–immune interactions rather than established determinants of thyroid disease or validated pharmacogenomic markers. This distinction is important because it prevents genetic association findings from being overinterpreted as evidence that vitamin D supplementation will produce different therapeutic effects according to VDR genotype. Further adequately powered, prospective, multi-ethnic and mechanistically integrated studies are required to establish whether VDR variation has clinically meaningful effects on thyroid autoimmunity, disease activity, or response to vitamin D-based interventions.

The VDR gene has several regions that have been most frequently studied including the FokI/rs2228570, BsmI/rs1544410, ApaI/rs7975232 and TaqI/rs731236 [98]. A meta-analysis investigating VDR gene polymorphisms and HT reported that the FokI polymorphism, but not the TaqI, ApaI, or BsmI polymorphisms, was significantly associated with HT [29]. Further evidence indicates that several VDR gene polymorphisms have been investigated for their potential association with autoimmune thyroid diseases [60,61]. In particular, the meta-analysis by Gao and Yu identified associations between several VDR polymorphisms and autoimmune thyroid disease, although these associations varied according to the specific autoimmune thyroid disease and the ethnicity of the study population [30]. The potential immunological role of vitamin D in HT has also been reported by Pakosiński et al. [31]. VDR gene polymorphisms may alter the structure, activity, or expression of the VDR and, consequently, modulate vitamin D-mediated immune responses, potentially contributing to the pathogenesis or susceptibility to HT.

However, it is essential to remain cautious in interpreting genetic findings in case-control studies. The main challenges include population structure, linkage disequilibrium and differences in exposure to environmental factors. Importantly, a single susceptibility polymorphism is unlikely to be used as a pharmacogenomic biomarker to predict response to vitamin D supplementation in patients with HT. Although several studies have investigated the association between VDR gene polymorphisms and autoimmune thyroid diseases, future studies are required to assess the effects of VDR gene variants on treatment responses in thyroid diseases. Prospective clinical trials are required to assess the effects of genotypes on clinical outcomes, including levels of thyroid autoantibodies, thyroid function, and clinical manifestations in patients with autoimmune thyroid diseases.

### 3.8. Vitamin D Metabolism, CYP27B1, CYP24A1 and Functional Vitamin D Resistance

**Figure 3:**
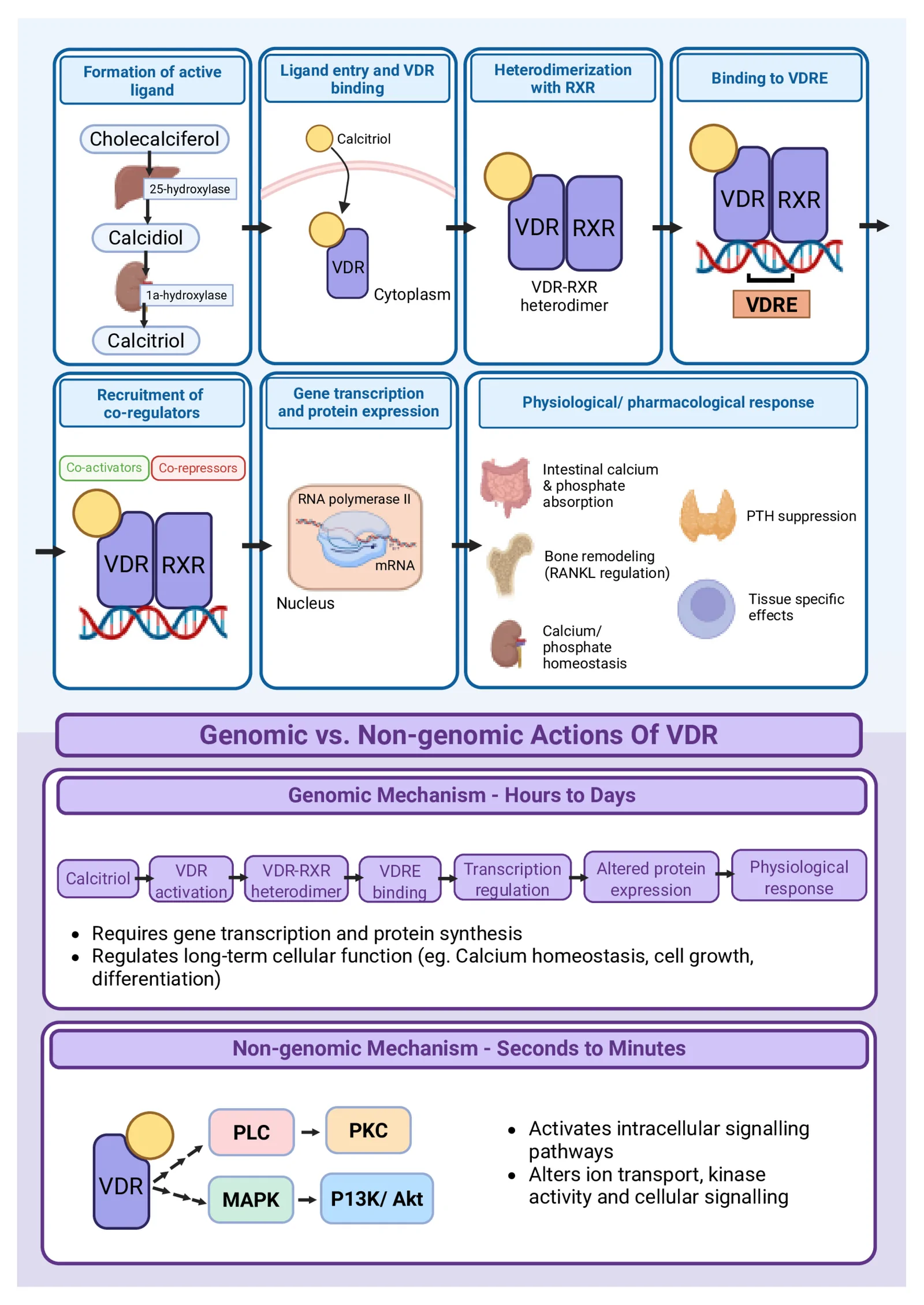
Genomic and non-genomic mechanisms of vitamin D–VDR signaling. Abbreviations: Akt, protein kinase B; MAPK, mitogen-activated protein kinase; PI3K, phosphoinositide 3-kinase; PLC, phospholipase C; PKC, protein kinase C; RXR, retinoid X receptor; VDR, vitamin D receptor; VDRE, vitamin D response element. Created in BioRender. Satyam, D. S. M. (2026) https://BioRender.com/xw6cks0.

Circulating 25(OH)D is the principal clinical indicator of vitamin D status; however, it does not fully reflect intracellular vitamin D activity. Vitamin D signaling depends on a multistep pathway involving transport and cellular uptake of vitamin D metabolites, local conversion of 25(OH)D to the biologically active hormone [1,25(OH)2D] by CYP27B1, binding of 1,25(OH)2D to the VDR, and subsequent VDR-dependent transcription [15,16]. CYP24A1 provides a major counter-regulatory pathway by catalyzing the inactivation and catabolism of 1,25(OH)2D and other vitamin D metabolites [108,109]. Thus, tissue-specific vitamin D activity is determined not only by circulating 25(OH)D concentrations but also by the balance between local activation, catabolism, transport, receptor abundance, and downstream transcriptional competence [110]. The canonical genomic and rapid non-genomic mechanisms through which activated VDR translates vitamin D availability into cellular and physiological responses are summarized in Figure 3.

Figure 3 illustrates the canonical genomic mechanism, in which calcitriol activates VDR, promotes VDR–RXR heterodimer formation, binding to VDREs, recruitment of transcriptional coregulators, and regulation of target-gene transcription. Vitamin D is generated through sequential hydroxylation of cholecalciferol to 25-hydroxyvitamin D [25(OH)D] and subsequently to the biologically active metabolite 1,25-dihydroxyvitamin D [1,25(OH)_2_D] (calcitriol), primarily through CYP2R1/CYP27B1-dependent metabolism. Calcitriol enters target cells and binds VDR, inducing conformational changes that facilitate heterodimerization with retinoid X receptor (RXR). The VDR–RXR complex binds vitamin D response elements (VDREs) within regulatory regions of target genes and recruits coactivator or corepressor complexes, thereby modifying chromatin accessibility and transcriptional activity. Subsequent changes in gene expression and protein abundance mediate longer-term effects on calcium and phosphate homeostasis, parathyroid hormone regulation, immune responses, cellular differentiation, proliferation, and other tissue-specific functions. This figure also depicts rapid non-genomic signaling associated with VDR-dependent activation of intracellular kinase and second-messenger pathways. Rapid non-genomic vitamin D responses can occur through membrane-associated VDR-associated signaling mechanisms involving phospholipase C/protein kinase C (PLC/PKC), mitogen-activated protein kinase (MAPK), and phosphoinositide 3-kinase/protein kinase B (PI3K/Akt) pathways. These rapid signaling events can influence ion transport, kinase activity, and cellular signaling independently of the slower transcription-dependent genomic response. The relative contribution of genomic and non-genomic mechanisms is tissue- and context-dependent.

CYP27B1 is therefore particularly important because local production of 1,25(OH)2D allows tissues to regulate vitamin D signaling independently of circulating concentrations. Although the kidney is a major site of systemic 1,25(OH)2D production, CYP27B1 is also expressed in several extrarenal tissues, including immune and endocrine tissues, where local synthesis may contribute to paracrine or intracrine vitamin D actions. This distinction is relevant to thyroid disease because vitamin D signaling within thyroid tissue may not be adequately represented by serum 25(OH)D alone [111,112]. Experimental studies of thyroid tissue have demonstrated expression of VDR, CYP27B1 and CYP24A1, with alterations in their expression across benign and malignant thyroid lesions, supporting the presence of a local vitamin D metabolic and signaling system within the thyroid [4,113].

CYP24A1 represents the principal metabolic mechanism limiting the duration and magnitude of vitamin D signaling. Its induction increases the conversion of active vitamin D metabolites toward inactive products and therefore acts as an important negative-feedback mechanism within the vitamin D endocrine axis [114,115]. Dysregulated CYP24A1 expression has been implicated in several chronic inflammatory, metabolic, malignant, and immune-mediated conditions, raising the possibility that increased local catabolism could reduce vitamin D signaling even when circulating 25(OH)D concentrations appear adequate [116]. Evidence from inflammatory and chronic disease models indicates that alterations in vitamin D hydroxylase expression can change local availability of active vitamin D and thereby uncouple circulating vitamin D status from tissue-level biological responses [117,118].

This concept provides a potential framework for understanding what may be termed functional vitamin D resistance. In this context, resistance does not necessarily imply absolute vitamin D deficiency; rather, it refers to reduced biological responsiveness to available vitamin D resulting from increased catabolism, impaired activation, altered transport, reduced VDR expression or function, or impaired downstream transcriptional signaling. CYP24A1-mediated degradation represents one potential mechanism, whereas abnormalities in VDR abundance or activity represent another [119]. Genetic variation in CYP24A1, CYP27B1, VDR and other components of the vitamin D pathway can further modify circulating vitamin D metabolites and tissue responsiveness, providing a potential explanation for inter-individual differences in response to similar vitamin D exposure.

Regulation of VDR itself provides an additional layer of complexity. VDR is a ligand-activated transcription factor whose biological activity depends on recruitment of coactivators and corepressors and on the chromatin environment of target genes. Silencing mediator of retinoid and thyroid hormone receptors (SMRT), for example, can interact with unliganded VDR and contribute to repression of VDR-responsive genes. Experimental studies have demonstrated that SMRT participates in repression of VDR target genes, indicating that receptor abundance alone does not completely determine transcriptional responsiveness [120]. SNAIL-family transcription factors provide another example of transcriptional regulation capable of reducing vitamin D responsiveness. SNAIL1 has been shown to repress VDR expression and reduce cellular responsiveness to 1,25(OH)2D, while SNAIL2 can further suppress VDR transcription and downstream vitamin D signaling [121]. These observations have been demonstrated predominantly in cancer models and should therefore be regarded as mechanistic evidence rather than direct evidence of VDR resistance in thyroid disease.

The distinction between circulating vitamin D sufficiency and tissue-level vitamin D activity is particularly relevant when interpreting studies of thyroid disease. Two individuals with similar serum 25(OH)D concentrations may theoretically have different biological responses because of differences in CYP27B1-mediated activation, CYP24A1-mediated catabolism, vitamin D-binding protein, VDR abundance or genetic variation, receptor co-regulators, and downstream transcriptional machinery. Studies have identified variants across several vitamin D pathway genes, including CYP2R1, CYP27B1, CYP24A1, VDR and genes encoding vitamin D transport proteins, that can influence circulating vitamin D metabolites or associations with non-skeletal phenotypes [101,122,123]. Consequently, serum 25(OH)D should be interpreted as an important but incomplete surrogate of vitamin D biological activity.

This framework has important implications for studies investigating vitamin D in autoimmune and non-autoimmune thyroid disease. A low serum 25(OH)D concentration may indicate inadequate substrate availability, whereas an apparently adequate concentration accompanied by limited biological response could reflect altered activation, increased catabolism, impaired receptor signaling, or other tissue-specific mechanisms. Conversely, an association between low 25(OH)D and thyroid autoimmunity does not establish that impaired VDR signaling is responsible unless intracellular metabolism and downstream activity are also evaluated. Thus, future thyroid studies should move beyond measurement of serum 25(OH)D alone and, where feasible, incorporate mechanistic markers such as 1,25(OH)_2_D, 24,25(OH)_2_D, CYP27B1 and CYP24A1 expression or activity, VDR expression, relevant VDR polymorphisms, and downstream VDR-responsive genes. Measurement of vitamin D-binding protein and free or bioavailable vitamin D may provide additional information in selected populations.

Such an approach may be particularly informative in chronic inflammatory thyroid disorders, in which altered immune signaling could theoretically influence local vitamin D metabolism and VDR responsiveness. However, direct evidence demonstrating that inflammation-induced CYP24A1 activation or transcriptional repression of VDR produces clinically meaningful vitamin D resistance specifically in Hashimoto’s thyroiditis or Graves’ disease remains limited. Therefore, the concept should currently be regarded as a mechanistic hypothesis supported by broader experimental and disease literature rather than an established clinical phenotype of thyroid disease. Establishing this relationship will require longitudinal and mechanistic studies integrating circulating vitamin D metabolites with tissue-level expression of CYP27B1, CYP24A1 and VDR, functional VDR-responsive biomarkers, genetic variation, and clinically relevant thyroid outcomes. This distinction could help determine whether apparently inconsistent associations between vitamin D status and thyroid disease reflect differences in circulating vitamin D exposure or, alternatively, differences in intracellular vitamin D metabolism and signaling. Table 2 summarises experimental, tissue-level and molecular evidence defining vitamin D–VDR signaling in thyroid biology.

### 3.9. Metabolic Disease as an Effect Modifier

The association between vitamin D status and thyroid disease should also consider the metabolic health of the participants studied. Vitamin D deficiency is frequent in subjects with thyroid diseases and many of them also present with other metabolic disturbances, such as obesity, insulin resistance, impaired glucose tolerance, and type 2 diabetes, and/or with lipid and other metabolic dysfunction-associated diseases. Circulating 25(OH)D levels are influenced by body adiposity and, in opposite ways, by metabolic health. Obesity, for example, affects both vitamin D levels and thyroid function and it is related to low-grade chronic inflammation and to changes in dietary habits and outdoor activities of obese individuals [130,131]. Moreover, adipose tissue is an endocrine organ that affects energy homeostasis, insulin sensitivity, and immune functions, and is involved in the modulation of TSH secretion [132]. Vitamin D deficiency is very common in individuals with thyroid dysfunction, and it is also frequently present in obese and diabetic subjects and in patients with autoimmune endocrine diseases [133,134].

The relation between vitamin D status and thyroid disease could be affected by several aspects of obesity. First, vitamin D is a fat-soluble hormone stored in adipose tissue. Hence, obese individuals might have higher storage of vitamin D and correspondingly lower levels of bioavailable vitamin D. In addition, adipose tissue is an endocrine organ that secretes adipokines and produces pro-inflammatory mediators. These can affect insulin sensitivity and immune function, and in turn affect the hypothalamic–pituitary–thyroid axis. There is evidence that increased adiposity is associated with changes in TSH levels in euthyroid subjects [135,136,137]. Moreover, insulin resistance with subsequent hyperinsulinemia could modulate thyroid hormone metabolism and the thyroid axis [138,139]. In turn, abnormal thyroid function could influence body weight as well as lipid and glucose metabolism and energy expenditure. Therefore, in the analysis of the relation between vitamin D and thyroid disease, body mass index, waist circumference, metabolic status, diet, and physical activity should be considered as confounders and effect modifiers.

Longitudinal data also warrant consideration of changes in body composition over time, and how they in turn affect vitamin D and thyroid function. For example, studies have found that weight loss is able to increase vitamin D levels, with changes in TSH also occurring over time [140,141,142]. While an increase in 25(OH)D levels may potentially affect thyroid function, it is essential to note that weight loss is also able to alter many factors, including insulin sensitivity, levels of inflammatory markers and adipokines, energy metabolism, and thyroid axis regulation [143,144,145]. As a result, longitudinal studies would benefit from the assessment of changes in body weight and adiposity, as well as serial measurement of 25(OH)D, TSH, free T4, free T3, glucose, insulin, HbA1c, and lipid profiles.

An important consideration in the relationship between vitamin D status and thyroid disease is the impact of metabolic disease on this relationship, including the effect on thyroid autoimmunity. Among the clinical outcomes investigated, reductions in TPOAb and TgAb concentrations represent a recurring favorable biochemical finding in some supplementation studies. Nevertheless, differences in study design, baseline vitamin D status, treatment regimens, and outcome assessment preclude the conclusion that these reductions occur consistently across studies.

One of the studies reported a relationship between vitamin D status and serum TRAb concentrations in patients with Graves’ disease [48]. In this longitudinal analysis including 171 patients with newly diagnosed Graves’ disease, patients with average baseline 25(OH)D concentrations of 20–29 ng/mL had higher rates of TRAb remission and also higher rates of negative conversion of TRAb during ATD treatment compared to patients with average baseline 25(OH)D below 20 ng/mL. Although this relationship seemed to be linear for negative conversion of TRAb, the relationship was non-linear for remission of TRAb. Importantly, however, no evidence was found that increasing 25(OH)D by vitamin D supplementation would result in better or even in similar outcomes of Graves’ disease.

Obesity and related metabolic diseases are not simply comorbid conditions but rather potential effect modifiers and confounders that should be considered in the analyses of the relationship between vitamin D status and thyroid diseases. Future studies should take into account adiposity and related metabolic phenotypes (BMI, waist circumference, visceral fat area, glucose and insulin metabolic profiles including HbA1c, cholesterol, lipid and triglycerides profiles, blood pressure, levels of physical activity, intakes of dietary vitamin D and calcium) as well as relevant drugs in their study designs and statistical models. Moreover, serial measurements of vitamin D and thyroid function tests would be more accurate than single measurements at baseline.

The relationship between vitamin D, VDRs and HT or Graves’ disease and their coexistence with metabolic diseases like type 2 diabetes, obesity and other associated conditions needs to be thoroughly investigated in longitudinal studies. In the clinical setting patients with HT or Graves’ disease should be evaluated in the frame of their metabolic status. Measurement of body composition, intake of nutrients, physical activity as well as measurement of glucose and lipid metabolism including vitamin D levels could give a more accurate interpretation of the relationships found in observational studies. The correction of clinically relevant deficiency states of vitamin D is supported by evidence, but there is currently not enough data to support the use of vitamin D supplements to prevent thyroid diseases, to induce remission or to affect long-term outcomes in patients with HT or Graves’ disease.

### 3.10. Pediatric Thyroid Disease and Skeletal Outcomes

Since pediatric patients continue to grow and mature until the end of adolescence, studies of this age group must consider all the factors that are associated with development of skeletal mass in children and adolescents. In pediatric patients with autoimmune thyroiditis, serum concentrations of 25(OH)D have been found to be decreased and the prevalence of vitamin D deficiency has been reported to be increased when compared to healthy children [146,147]. In one study, children with HT were found to have a frequency of vitamin D deficiency that was substantially higher than that of children without autoimmune thyroid disease [147]. In addition, serum 25(OH)D concentrations were found to be inversely correlated with levels of anti-thyroid peroxidase antibodies. A second study found decreased concentrations of 25(OH)D in children and adolescents with autoimmune thyroiditis. The study also found that 25(OH)D concentrations were inversely correlated with levels of anti-TPO and anti-thyroglobulin antibodies as well as with TSH concentrations. However, in multivariate analysis, vitamin D status was not found to be an independent risk factor for the progression to hypothyroidism [148]. The findings of these studies suggest an association between vitamin D status and pediatric autoimmune thyroid disease. However, they do not establish whether vitamin D deficiency contributes to disease development, occurs because of the disease, or reflects shared confounding factors, such as increased adiposity, poor dietary habits, and reduced outdoor activity and sunlight exposure.

The well-known effects of thyroid hormone on bone in adults are of less relevance to children with thyroid disease. This is because children of any age are still in growth and hence are laying bone at varying rates depending on their age, sex, and size. As bone is forming, the Bone mineral density (BMD) of children with untreated hyperthyroidism has been reported to be low for their age and size in up to 40% of cases with mean values for lumbar spine, femoral neck and total body BMD below that of age- and sex-matched controls [149]. A significant inverse correlation has, however, been found between serum-free thyroid hormone and lumbar spine BMD in children with hyperthyroidism [150,151]. Markedly increased bone turnover with a characteristic pattern of elevated serum alkaline phosphatase (ALP) and low bone ALP/total ALP ratio is also typical of children with untreated, hyperthyroidism due to Graves’ disease and, almost certainly, other causes of excessive thyroid hormone [151]. The changes in BMD described above for children with Graves’ disease return to normal within 1–2 years of achieving a euthyroid state. Thus, excess thyroid hormone itself appears to have a marked adverse effect on the skeleton of children with thyroid disease and, therefore, must be regarded as a confounder variable of potential importance in the assessment of the independent and clinical relevance of vitamin D status to bone in this setting.

Children grow and reach mature height within a relatively short time span, and, during this time, the development of bone mass occurs. To accurately interpret the results of BMD measurements in children, the results must be expressed in terms of Z-scores. Z-scores provide a measure of a child’s BMD in relation to other children of the same age, sex, and size (or bone length). Children with similar Z-scores within a specified range have similar quantities of bone. Although a single measurement of BMD provides only a ‘snapshot’ or cross-sectional view of the child’s bone status, BMD in children changes continuously over time and, typically, increases with age. By reassessing BMD in children at regular time intervals, it is possible to assess whether a child with delayed mineralization is still accumulating bone at a normal rate. Importantly, other factors that change over time in a child with autoimmune thyroiditis (such as thyroid hormone levels, therapy, growth rate, and age at puberty) could affect BMD. For this reason, it is essential to take account of all these factors when assessing BMD in children with thyroid diseases.

Additional information on the role of vitamin D in determining bone health can be gained from studies of otherwise healthy children and adolescents. In cross-sectional analyses, higher levels of 25(OH)D have been associated with increased bone mineral content and density. A few randomized studies of vitamin D supplementation in vitamin D deficient children and adolescents have also investigated the effects of intervention on bone health. However, the degree to which serum 25(OH)D influences bone in pediatric thyroid disease is likely to vary according to several factors including the child’s age and the developmental stage at which intervention takes place. Studies of vitamin D status in children with thyroid disease therefore need to take account of vitamin D status and other determinants of bone health, including measurements of thyroid function, calcium and phosphate metabolism and bone turnover.

Most of the studies on the relationship between vitamin D and thyroid disease have been cross-sectional. To get a better understanding of the current situation in pediatric thyroid disease a longitudinal study is necessary. In this type of study, children with thyroid disease and controls would be studied at several time points. Vitamin D, TSH, free T4/free T3, thyroid autoantibodies, calcium, phosphate, PTH, height, growth rate, pubertal status and skeletal parameters would be measured at each time point. In addition, BMD could be expressed as a Z-score, using the age- and size-adjusted means of healthy children. Of note, potential confounders such as growth hormone/Insulin-like growth factor 1 (IGF-1) signaling, puberty, body composition, diet with regards to calcium and vitamin D intake, and physical activity as well as the duration of treatment would have to be considered. VDR polymorphisms and, where available, measures of VDR expression or vitamin D metabolic pathways may provide additional mechanistic information regarding inter-individual differences in vitamin D responsiveness.

Thyroid disease in children and adolescents is a distinct clinical entity. The evaluation of the contribution of vitamin D/VDR signaling to the effects of vitamin D on skeleton and thyroid in pediatric thyroid disease needs to consider the complex interaction of vitamin D status, thyroid hormone status, and thyroid autoimmunity in children and adolescents going through growth and skeletal maturation. Thus, longitudinal studies measuring vitamin D status, thyroid function and autoimmunity, growth and pubertal development, and bone and calcium metabolism over time are more appropriate to study the effects of vitamin D on skeleton and thyroid in pediatric thyroid disease and to assess the benefits of correction of vitamin D deficiency for skeletal and thyroid health in children and adolescents.

### 3.11. Thyroidectomy, Radioactive Iodine and Calcium-Phosphate Homeostasis

The perioperative period represents a time during which total thyroidectomy (and other thyroid surgery) affects calcium homeostasis and decreases vitamin D status. Vitamin D status is clinically relevant in patients undergoing thyroidectomy because adequate vitamin D availability supports intestinal calcium absorption and calcium homeostasis [72,152,153]. Postoperative hypocalcemia, however, is multifactorial and is strongly associated with transient or permanent impairment of parathyroid function, including inadvertent parathyroid injury, devascularization, or removal. Preoperative vitamin D deficiency may represent an additional risk factor in some populations, but it should not be considered the sole cause of postoperative hypocalcemia. Evidence concerning preoperative supplementation suggests a potential role in optimizing calcium homeostasis; its preventive effectiveness should be interpreted considering the available trial evidence and the clinical context [42,72,73]. Patients with vitamin D deficiency are at higher risk of developing hypocalcemia after thyroid surgery, with a study concluding that preoperative supplementation can reduce this risk [42]. The thyroid surgery cohorts have been reported to be an important clinically relevant group [42,154,155]. The Metabolic and associated endocrine effects of Radioactive iodine (RAI) (in the form of declines in PTH and resulting decreases in calcium and phosphorus levels with stable 25(OH)D) are also reported and suggest that the cause of hypocalcemia after RAI is primarily due to dysfunction of the parathyroid glands [156,157]. Baseline levels of vitamin D appear to influence severity in such patients. Studies of patients treated with RAI have demonstrated a decline in PTH, calcium and phosphorus in the immediate period following RAI. Importantly, levels of 25(OH)D remained stable. Hence, while changes in vitamin D status may, in part, contribute to the changes in calcium metabolism seen following RAI, these are likely to be of greater importance as an acute phenomenon and are due to the dysfunction of the parathyroid glands. However, the influence of pre-treatment vitamin D status may, in addition, affect the severity of the calcium-related disturbances encountered by patients following RAI.

## 4. Discussion

This review indicates that the relationship between vitamin D–vitamin D receptor (VDR) signaling and thyroid disorders is heterogeneous and depends on the underlying phenotype, study design, and clinical context. Observational studies frequently report lower serum 25-hydroxyvitamin D concentrations in patients with autoimmune thyroid disease, particularly Hashimoto’s thyroiditis (HT), although findings are not uniform across populations [62,80,88,133]. Differences in analytical methods, definitions of vitamin D deficiency, baseline vitamin D status, season, age, adiposity, supplementation, and adjustment for confounding factors limit direct comparisons between studies. Furthermore, the direction of the association remains uncertain because low vitamin D status may precede autoimmune disease, arise because of disease-related changes, or reflect shared determinants such as obesity, inflammation, physical inactivity, and comorbidities. Although some intervention studies report reductions in thyroid autoantibody concentrations following vitamin D supplementation [88,158]. These findings concern immunological surrogate outcomes and do not establish improved thyroid function, prevention of overt hypothyroidism, or sustained clinical benefit. Consistent with this distinction, the D-Health trial did not demonstrate a general preventive effect of monthly vitamin D supplementation on incident hypothyroidism [44]. Overall, the evidence supports a possible modulatory role for vitamin D in thyroid-related immune and metabolic processes but does not establish vitamin D deficiency as a universal cause of thyroid dysfunction or justify routine supplementation specifically for thyroid disease prevention or treatment.

The clinical interpretation differs between HT, Graves’ disease (GD), and non-autoimmune thyroid dysfunction. HT involves immune-mediated thyroid follicular injury and is commonly associated with thyroid peroxidase antibodies (TPOAb) and thyroglobulin antibodies (TgAb), whereas GD is characterized by thyrotropin receptor antibodies (TRAb) that can stimulate thyroid hormone production [38,94,95,96,97,98,159]. Vitamin D–VDR signaling can influence antigen presentation, T-cell differentiation, regulatory T-cell activity, B-cell function, and inflammatory cytokine pathways, providing biological plausibility for its involvement in thyroid autoimmunity [160,161,162]. However, mechanistic plausibility does not establish causation or clinical efficacy. An open-label randomized controlled trial reported a reduction in TPOAb following vitamin D supplementation in patients with autoimmune thyroid disease [88,163]. Other studies have investigated vitamin D status in specific populations, including premenopausal women and women with polycystic ovary syndrome, while retrospective evidence has examined vitamin D deficiency in autoimmune thyroiditis [164,165,166]. These findings should be interpreted in relation to their respective populations and study designs rather than generalized to all women or all autoimmune thyroid disorders. Dutta et al. provides broader context concerning vitamin D, thyroid autoimmunity, and cancer but this should not substitute for primary studies when specific clinical outcomes are discussed [167]. Importantly, reductions in thyroid autoantibody concentrations do not necessarily indicate preservation of thyroid tissue, normalization of thyroid function, reduced medication requirements, or prevention of disease progression. For GD, remission, relapse, thyroid function, and treatment requirements are more clinically informative than TRAb changes alone. In non-autoimmune hypothyroidism caused by substantial or irreversible loss of functional thyroid tissue, a proposed immunomodulatory effect of vitamin D would not be expected to restore lost hormone-producing capacity.

Potential interactions between thyroid hormone signaling and the vitamin D–VDR pathway provide a further mechanistic context, although the evidence for direct reciprocal regulation in human thyroid disease remains limited. VDR and thyroid hormone receptors (TRα and TRβ) belong to the nuclear receptor superfamily and regulate transcription through ligand-dependent interactions with DNA response elements and transcriptional co-regulators [168]. Both receptor systems can interact with retinoid X receptor (RXR), providing a potential molecular basis for transcriptional crosstalk [169]. However, shared receptor partners and co-regulatory machinery do not, by themselves, establish direct functional competition or clinically important cross-regulation. Thyroid hormone status may influence vitamin D-related outcomes indirectly through changes in skeletal turnover, calcium–phosphate homeostasis, metabolic rate, body composition, and systemic physiology [82,170]. Hyperthyroidism increases bone turnover and may complicate the interpretation of circulating vitamin D concentrations in affected patients [79]. Conversely, VDR-dependent signaling may influence cellular differentiation, immune regulation, and gene-expression programs relevant to endocrine physiology [171,172]. Nevertheless, direct evidence that vitamin D supplementation consistently alters thyroid hormone receptor expression or receptor-mediated thyroid hormone action in patients with autoimmune thyroid disease is insufficient. Similarly, whether thyroid hormone receptors directly regulate vitamin D synthesis or catabolism in clinically relevant human tissues requires further investigation. Accordingly, the two receptor systems should be regarded as potentially interconnected components of endocrine regulation rather than as an established direct causal pathway in thyroid disease.

Vitamin D activity also depends on metabolic processes that are not fully captured by circulating 25(OH)D concentrations. CYP27B1 contributes to the conversion of 25(OH)D to biologically active 1,25-dihydroxyvitamin D, whereas CYP24A1 participates in the catabolism of vitamin D metabolites [15,16,108,109,110]. Consequently, local metabolite availability, tissue-specific enzyme activity, VDR expression, receptor function, and downstream transcriptional responses may influence biological activity despite similar circulating 25(OH)D concentrations. This distinction is particularly relevant when interpreting apparently inconsistent relationships between vitamin D status and thyroid outcomes. However, evidence that altered CYP27B1 or CYP24A1 activity produces clinically meaningful impairment of vitamin D signaling specifically in HT or GD remains limited [62]. Circulating vitamin D concentrations alone cannot establish the presence of tissue-level resistance, and molecular differences should not be interpreted as disease mechanisms unless supported by functional evidence and relevant clinical outcomes.

Genetic variation may contribute to interindividual heterogeneity in vitamin D signaling and thyroid autoimmunity, but current evidence does not establish individual variants as causal determinants or validated predictors of treatment response. A meta-analysis of 11 case-control studies found that the VDR FokI polymorphism was associated with HT in the overall analysis, with statistically significant associations in the Asian subgroup but not in the Caucasian subgroup. In contrast, TaqI, ApaI, and BsmI were not significantly associated with HT in the overall analysis [29]. The absence of statistical significance in the Caucasian subgroup does not establish that an association is absent; it indicates that the available subgroup evidence did not demonstrate statistical significance. The limited number of contributing studies, potential differences in statistical power, and population-specific genetic and environmental factors warrant cautious interpretation. These findings suggest possible genetic heterogeneity rather than proving a causal role for VDR polymorphisms in HT or GD. A study examining thyroid dysfunction and VDR polymorphisms in a population with keratoconus provides related but indirect evidence and should not be treated as direct confirmation of a genetic contribution to autoimmune thyroid disease [173].

Vitamin D resistance has also been proposed as a possible explanation for differences between circulating vitamin D concentrations and tissue-level biological responses [172,174,175]. Potential mechanisms include altered VDR expression or function, changes in intracellular vitamin D metabolism, differences in receptor co-regulators, and impaired downstream transcriptional responses. However, the hypothesis that vitamin D resistance contributes to autoimmune disease has not established that it causes hypothyroidism or explains the inconsistent relationship between circulating vitamin D status and thyroid function [175]. Vitamin D deficiency and vitamin D resistance should be distinguished-deficiency concerns inadequate vitamin D availability, whereas resistance implies reduced biological responsiveness despite the availability of the relevant ligand. These states may coexist but are not interchangeable and cannot be inferred from the same laboratory measurement alone. A lack of consistent association between serum 25(OH)D and thyroid function is not, by itself, evidence of resistance. Establishing this mechanism would require direct assessment of relevant metabolites, VDR function, downstream target-gene responses, or validated functional biomarkers. Until such evidence is available, vitamin D resistance should be regarded as a testable mechanistic hypothesis rather than an established cause of thyroid disease or a justification for high-dose supplementation.

Sex-related biological differences may further modify the relationship between vitamin D signaling and thyroid autoimmunity [37,176]. Autoimmune thyroid diseases are more common in women, and estrogen and androgen signaling can influence immune responses, body composition, skeletal physiology, and endocrine function [177,178,179]. Broader autoimmune-disease and experimental literature suggests potential interactions between sex hormones and vitamin D signaling, including possible effects on VDR expression and immune-cell responses [176,180,181]. However, these observations do not establish that vitamin D has a stronger or weaker causal effect on thyroid disease in women than in men. Findings from premenopausal women or women with polycystic ovary syndrome should not automatically be generalized to other populations [164,165]. A predominantly female study population does not, by itself, demonstrate a sex-specific association; such a conclusion requires adequately powered sex-stratified analyses or formal tests of interaction. Age, menopausal status, pregnancy or postpartum status, hormonal exposure, ethnicity, adiposity, diet, sunlight exposure, physical activity, inflammation, comorbidities, and medication use may all influence the observed relationship. In addition, thyroid dysfunction can itself alter body composition, energy expenditure, skeletal turnover, and calcium–phosphate metabolism, creating the possibility of reverse causality. Sex and hormonal status should therefore be investigated as potential effect modifiers rather than assumed to establish a sex-specific causal or therapeutic effect.

The clinical implications should remain proportionate to the strength of the evidence. Vitamin D supplementation is not a substitute for established treatment of hypothyroidism or GD, and current evidence does not support universal supplementation specifically to prevent or treat thyroid disease. Correction of documented vitamin D deficiency may nevertheless be appropriate for established nutritional and skeletal indications. Thyroidectomy represents a distinct clinical setting in which vitamin D status may be relevant to perioperative calcium management. Postoperative hypocalcemia is multifactorial and is strongly influenced by parathyroid injury or dysfunction. Optimizing vitamin D status may support calcium absorption and homeostasis, but it should not be assumed to prevent parathyroid damage or reliably prevent postoperative hypocalcemia [42]. Evidence concerning preoperative supplementation supports further evaluation rather than an unconditional claim of prevention. Future studies should determine whether vitamin D correction improves clinically relevant calcium outcomes when used alongside established perioperative assessment and management.

This review integrates observational, interventional, genetic, and experimental evidence across autoimmune and non-autoimmune thyroid conditions. Nevertheless, several limitations constrain interpretation. The included studies differed in design, participant characteristics, vitamin D assays, definitions of deficiency, baseline 25(OH)D concentrations, supplementation regimens, follow-up duration, and outcome definitions. This heterogeneity limits direct comparisons and complicates interpretation of the overall evidence. Much of the clinical evidence was observational and cannot establish causality; genetic associations may be population-specific, and experimental findings may not translate directly to human thyroid disease. The search was restricted to PubMed/MEDLINE and Scopus and to English-language publications, so relevant evidence may have been missed. In addition, no formal risk-of-bias assessment or certainty-of-evidence framework was applied.

Future research should prioritize adequately powered, phenotype-specific prospective studies and randomized controlled trials that distinguish HT, GD, non-autoimmune thyroid dysfunction, and treatment-related outcomes. Trials should assess clinically meaningful endpoints alongside antibody concentrations, account for baseline vitamin D status and relevant confounders, and include sufficient follow-up to determine whether any benefits are sustained. Mechanistic investigations should examine thyroid hormone receptor–VDR crosstalk, local vitamin D metabolism, VDR expression and function, and downstream transcriptional responses in appropriate human tissues or validated experimental models. Genetic studies should include sufficiently large and diverse populations to evaluate reproducibility and potential population differences. Investigations of vitamin D resistance and sex-hormone interactions should establish functional mechanisms and determine whether they predict meaningful differences in disease course or treatment response. Until such evidence is available, neither genetic nor molecular measures should be used routinely to guide individualized vitamin D supplementation for thyroid disease. Overall, vitamin D–VDR signaling is a biologically plausible component of the interaction among immune regulation, metabolism, skeletal physiology, and thyroid function, but its clinical significance remains phenotype dependent and is not yet sufficient to support thyroid-specific supplementation as a universal therapeutic strategy.

## 5. Conclusions

The evidence synthesized in this systematic review demonstrates that vitamin D–VDR signaling is biologically connected with thyroid disease through a complex network involving immune regulation, thyroid-cell biology, genetic variation, vitamin D metabolism, metabolic status and calcium-phosphate homeostasis. The relationship is nevertheless phenotype dependent. Lower vitamin D status is frequently observed in thyroid disease, particularly HT and other autoimmune phenotypes, and is repeatedly associated with higher thyroid autoantibody concentrations. Vitamin D supplementation may reduce TPOAb and TgAb concentrations in some patients with autoimmune thyroid disease, but the clinical significance of these changes remains uncertain. Available evidence does not establish that antibody reductions translate into improved thyroid function, prevention of overt hypothyroidism, reduced levothyroxine requirements, or sustained disease remission. Furthermore, randomized evidence does not support a general preventive effect of vitamin D supplementation on incident hypothyroidism in the populations studied. Vitamin D deficiency should be managed according to established clinical indications, without substituting supplementation for indicated thyroid-specific treatment.

These apparently divergent findings are best explained by a model in which vitamin D functions as a biological modifier rather than a universal determinant of thyroid disease. VDR signaling may be particularly relevant to immune-mediated thyroid pathology, whereas its contribution to non-autoimmune thyroid dysfunction is less established. VDR polymorphisms may influence susceptibility and potentially explain heterogeneity in response, but current evidence does not justify routine genotype-guided supplementation. CYP27B1, CYP24A1, VDR expression and epigenetic regulation provide additional mechanisms through which tissue-level vitamin D activity may differ despite similar serum 25(OH)D concentrations. The perioperative evidence introduces a distinct translational application in which vitamin D status may influence postoperative calcium homeostasis following thyroidectomy. This supports investigation of vitamin D optimization as a component of risk stratification while recognizing that parathyroid injury remains the principal proximal determinant of postoperative hypocalcemia.

The principal research opportunity emerging from this review is therefore to move beyond the concept of “vitamin D deficiency in thyroid disease” toward a functional vitamin D–VDR response phenotype incorporating serum 25(OH)D, vitamin D metabolism, VDR genotype, receptor expression, immune phenotype, thyroid autoantibodies, thyroid function, metabolic status and quantitative tissue biomarkers. Integration of disease-specific experimental models, advanced imaging and multimodal computational analysis could enable identification of patients most likely to respond to VDR-directed interventions. Thus, vitamin D should neither be regarded as a universal treatment for thyroid disease nor dismissed as a nonspecific nutritional correlate. The current evidence supports a more precise interpretation: the vitamin D–VDR axis represents a biologically credible regulatory interface whose therapeutic value is likely to depend on thyroid disease etiology, immune phenotype, metabolic context and receptor-level biology. Establishing this precision framework through adequately powered, genotype-informed and mechanistically anchored clinical trials represents the critical next step toward translation.

## Figures and Tables

**Table 1:** Selected human clinical, epidemiological, genetic, and thyroid tissue studies investigating vitamin D status and VDR-related findings in thyroid disease.

Study/Model	*n*	Thyroid Phenotype/Population	Vitamin D/VDR Component	Principal Finding	Evidence Interpretation
Randomized, double-blind placebo-controlled D-Health trial [44]	21,315 randomized; 17,851 main analysis	Australians ≥ 60 years; prevention of hypothyroidism	Monthly vitamin D3 60,000 IU	Vitamin D supplementation did not reduce incident hypothyroidism compared with placebo	Does not support prevention of hypothyroidism by intermittent high-dose vitamin D in this population
Short correspondence [45]	Not applicable	Thyrotoxicosis/hyperthyroidism	25(OH)D levels and vitamin D metabolism	Thyrotoxicosis may accelerate vitamin D metabolism, potentially lowering 25(OH)D and increasing the risk of vitamin D insufficiency or deficiency.	The letter summarizes previous observations and recommends monitoring vitamin D status in thyrotoxic patients; it does not provide new experimental or clinical evidence.
Prospective clinical study [46]	30 patients	Graves’ disease	Vitamin D and skeletal metabolism	During treatment of Graves’ disease, 25(OH)D decreased, 1,25(OH)_2_D and PTH increased, bone resorption decreased, and BMD improved.	Demonstrates altered vitamin D metabolism and bone metabolism during treatment of Graves’ disease; observational and does not establish that vitamin D changes caused the thyroid or skeletal outcomes.
Multicentre double-blind randomized placebo-controlled DAGMAR trial [47]	278 included; 274 after 4 withdrawals	Newly diagnosed Graves’ disease treated with antithyroid drugs	Vitamin D3 2800 IU/day vs. placebo	Vitamin D supplementation did not improve entry into or maintenance of remission	Important counterpoint to observational Graves’ disease findings in [48,49]
Retrospective observational [48]	171 recruited; 76 eligible for follow-up analysis	Newly diagnosed Graves’ disease	Baseline 25(OH)D and TRAb	Vitamin-D status was associated with TRAb remission/negative conversion during treatment	Observational association; causality cannot be inferred
Cross-sectional study [49]	Total 75; active GD 41, remission GD 34	Active versus remission Graves’ disease	Serum vitamin D	Active GD had a higher prevalence of vitamin-D deficiency; mean vitamin D was not significantly different	Suggests disease-state association rather than established causality
Systematic review/meta-analysis of RCTs [41]	8 RCTs; *n* = 652	Hashimoto’s thyroiditis	Vitamin-D supplementation	Vitamin D supplementation reduced TPOAb and TgAb titres	Intervention evidence for antibody outcomes, not necessarily thyroid-function normalization
Community-based prospective program/nested case-control analysis [50]	11,017	Participants in a health and wellness program; subgroup with hypothyroidism and euthyroid controls	Vitamin-D supplementation targeting physiological 25(OH)D	Increases in 25(OH)D accompanied decreases in TSH, thyroid antibodies and hypothyroidism prevalence over 12 months	Large observational/intervention-program dataset; absence of randomized allocation limits causal interpretation
Retrospective observational study [51]	256	Preconception women	Serum 25(OH)D and TPOAb	Lower 25(OH)D was independently associated with TPOAb positivity	Relevant reproductive-age autoimmune-thyroid evidence; observational
Cross-sectional study [52]	503	Adults with type 2 diabetes mellitus	25(OH)D, TPOAb/TgAb, thyroid hormones	TPOAb positivity was associated with lower 25(OH)D, whereas TgAb positivity showed an opposite relationship	Shows context-specific, directionally opposing associations in patients with T2DM.
Pediatric observational study [53]	Total 120: 54 GD, 36 HT, 30 controls	Children with autoimmune thyroid disease	25(OH)D and IL-21	25(OH)D was lower and IL-21 higher in GD/HT; vitamin D correlated negatively with IL-21 and thyroid antibodies	Links vitamin-D status with inflammatory immune phenotype
Large epidemiological survey [54]	1812	General Chinese population; thyroid autoimmunity/HT	25(OH)D, TPOAb, TgAb	Vitamin-D deficiency was associated with increased TPOAb and TgAb positivity; Th1/Th17-related abnormalities were also observed	Strong observational evidence linking vitamin-D status with thyroid autoimmunity
Comparative observational study [55]	Total 150: 50 AITD, 50 non-AITD, 50 controls	Primary hypothyroidism	25(OH)D, TSH, TPOAb/TgAb	Vitamin-D levels were lower in autoimmune hypothyroidism and related to thyroid-antibody status	Supports association between vitamin-D status and autoimmune hypothyroidism
Human observational genetic association study [56]	153 thyroid-pathology patients	Thyroid pathology in West-Ukrainian population	VDR rs2228570 and serum 25(OH)D	Investigated genotype-dependent differences in serum vitamin-D status	Reports subgroup-specific differences in 25(OH)D according to rs2228570 genotype, but does not establish genotype as a general risk factor for vitamin-D deficiency.
Meta-analysis [57]	1820 GD cases; 2066 controls	Graves’ disease	VDR ApaI, BsmI, TaqI and FokI	Associations differed substantially by ethnicity, with stronger signals reported in Asian populations	VDR polymorphisms may contribute to GD susceptibility in Asians, but evidence differs by ethnicity.
Human case-control genetic association study [58]	121 AITD patients; 117 controls	Adults with autoimmune thyroid diseases: 16 GD and 105 HT patients	Genotyping of VDR FokI (rs2228570) and ApaI polymorphisms using PCR-RFLP; no vitamin D supplementation or serum 25(OH)D measurement.	FokI genotypes showed associations with AITD/HT in adjusted analyses; ApaI did not show a significant association	Genetic evidence linking VDR variation with autoimmune thyroid disease susceptibility, but observational and population-specific.
Human case–control observational genetic/biochemical study [59]	222 participants:116 AITD; 106 controls	17 GD and 99 HT	Serum vitamin D and VDR FokI/ApaI/BsmI/TaqI	AITD patients had lower serum vitamin D than controls, mainly driven by HT; VDR polymorphisms were not associated with AITD overall, but FokI was associated with GD	Vitamin D deficiency was associated with AITD, particularly HT, while FokI showed a subtype-specific association with GD. The GD subgroup was only 17 patients, so the FokI finding should be interpreted cautiously and is described by the authors as requiring replication.
Human observational genetic association study [60]	Total 331: 139 GD, 116 HT, 76 controls	Autoimmune thyroid disease (Graves’ disease and Hashimoto’s disease)	Genotyping of VDR, GC and CYP2R1 polymorphisms	VDR polymorphisms were associated with AITD development and/or GD activity, while GC and CYP2R1 polymorphisms were associated with GD intractability	Supports pathway-level rather than single-gene genetic effects
Meta-analysis [61]	8 eligible publications comprising 10 distinct study populations and a total of 3403 participants (1708 cases and 1633 controls)	Autoimmune thyroid disease	VDR FokI, TaqI, BsmI and ApaI	Pooled evidence supported associations of BsmI and TaqI with autoimmune thyroid disease, whereas ApaI and FokI were not significant overall; subgroup effects varied by population.	Evidence for genetic heterogeneity rather than a universal VDR effect
Human thyroid tissue-based comparative study [4]	Snap-frozen tissue from 64 patients (RT-PCR); paraffin-embedded tissue from 72 patients (immunohistochemistry)	Normal thyroid, follicular adenoma (FA), differentiated thyroid cancer, anaplastic thyroid cancer (ATC)	VDR, CYP27B1, CYP24A1	VDR, CYP27B1 and CYP24A1 expression varied by tumor type and stage; VDR was reduced in aggressive/anaplastic disease.	Supports stage-dependent alterations in local vitamin D signaling.
Retrospective clinical + thyroid-tissue transcriptomic study [62]	197 clinical patients: 87 HT, 110 NT; 18 tissues for RNA-seq	Hashimoto’s thyroiditis	Serum vitamin D, VDR and vitamin-D-related transcriptome	Serum vitamin D was lower in HT, whereas VDR and vitamin-D-related pathways were upregulated in thyroid tissue	Particularly important evidence for a distinction between circulating vitamin-D status and tissue-level VDR activity

Abbreviations: AITD, autoimmune thyroid disease; GD, Graves’ disease; HT, Hashimoto’s thyroiditis; NT, non-Hashimoto thyroid tissue/control group; 25(OH)D, 25-hydroxyvitamin D; CYP27B1, cytochrome P450 family 27 subfamily B member 1; CYP24A1, cytochrome P450 family 24 subfamily A member 1; TPOAb, thyroid peroxidase antibody; TgAb, thyroglobulin antibody; TRAb, thyrotropin receptor antibody; TSH, thyroid-stimulating hormone; VDR, vitamin D receptor. Table 1 presents 21 studies intended to illustrate key human evidence domains, principal findings, and relevant limitations; they do not constitute an exhaustive listing of all 118 primary studies included in the qualitative synthesis.

**Table 2:** Selected experimental, mechanistic and thyroid specific studies investigating vitamin D–VDR signaling in thyroid-related biological processes.

Study Type	Brief Study Description	Outcome Measures	Principal Findings	Direct Thyroid Evidence
Review of experimental and thyroid literature [3]	Human and experimental thyroid literature	1,25(OH)_2_D–VDR signaling, VDR, CYP27B1, CYP24A1 and vitamin-D effects in normal thyroid and thyroid cancer	Impaired 1,25(OH)_2_D–VDR signaling may contribute to thyroid cancer development and progression, while preclinical studies suggest potential antitumor effects of vitamin D analogues, particularly in advanced thyroid cancer.	No; Indirect/secondary
Human tissue-based comparative study [4]	Snap-frozen tissue from 64 patients (RT-PCR); paraffin-embedded tissue from 72 patients (immunohistochemistry)	VDR, CYP27B1 and CYP24A1 expression assessed by RT-PCR and immunohistochemistry	VDR, CYP27B1 and CYP24A1 were increased in benign and differentiated thyroid tumors; VDR was often lost in ATC, with heterogeneous changes in CYP27B1/CYP24A1.	Yes
In vitro molecular/cell-based experimental study [120]	Experiments were performed mainly in HEK293 human embryonic kidney cells	SMRT–VDR interaction	SMRT directly mediates transcriptional repression by unliganded VDR through SMRT-ID1, with calcitriol relieving this repression.	No; general mechanistic evidence
Cancer-cell molecular model [121]	Colon cancer cell models were used, together with human colorectal tumor RNA-expression data to examine SNAI1, SNAI2, and VDR expression.	VDR expression in relation to SNAIL1/SNAIL2	SNAIL-family transcription factors suppress VDR expression and vitamin-D responsiveness	No; non-thyroid cancer model
Human thyroid tissue + RNA-seq [62]	197 clinical patients: 87 HT and 110 without HT; thyroid tissue obtained from 37 patients; 18 tissues underwent RNA-seq	Obtained thyroid tissue was used for VDR and vitamin-D pathway genes analysis	HT was associated with lower serum vitamin D but increased tissue VDR expression and vitamin-D-related pathway activity	Yes
Animal experimental model of Graves’ hyperthyroidism [124]	BALB/c and C57BL/6 mice were studied under vitamin-D-deficient or sufficient conditions.	Vitamin-D-deficient vs. sufficient diet with TSH-receptor immunization; 25(OH)D, 1,25(OH)_2_D and Cyp27b1 assessed.	Vitamin-D deficiency produced relatively minor changes in TSH-receptor immune responses but was associated with lower baseline T4 and more persistent hyperthyroidism in BALB/c mice	Yes
Experimental autoimmune thyroiditis in rats [125]	48 female Wistar rats	1,25(OH)_2_D administered prophylactically or therapeutically; thyroid histology, thyroid autoantibodies and cytokines assessed	1,25(OH)_2_D reduced autoimmune/inflammatory thyroid changes and modulated the Th1/Th2 cytokine profile	Yes
Animal metabolic/endocrine model [126]	30 male Wistar rats	Streptozotocin-induced diabetes ± vitamin D3; FT3, FT4, TSH and DIO2 expression assessed	Vitamin D3 improved diabetes-associated thyroid hormone abnormalities and increased DIO2 expression in peripheral tissues	No; Indirect/secondary
Human thyroid-tissue study [127]	35 patients with PTC; 35 primary tumors and 9 lymph-node metastases for IHC; frozen tumor/normal tissue for RT-qPCR	VDR and CYP27B1/1α-hydroxylase expression assessed by RT-qPCR and immunohistochemistry	VDR and CYP27B1 were expressed in PTC; their expression pattern varied with tumor stage/metastatic tissue, supporting altered local vitamin-D signaling in PTC	Yes
Thyroid-cancer cell/stem-like-cell model [128]	ATC cell lines and thyrosphere-derived thyroid cancer stem-like cells	Calcitriol treatment; proliferation, sphere formation, cell cycle and apoptosis assessed	Calcitriol inhibited proliferation and sphere formation of thyroid cancer stem-like cells and promoted cell-cycle arrest, supporting an antiproliferative role of vitamin-D signaling	Yes
Anaplastic thyroid-cancer cell model [129]	ATC cell lines 8305C and 8505C	1,25(OH)_2_D and vitamin-D analog MART-10	Calcitriol and MART-10 reduced migration/invasion and altered EMT-associated pathways; MART-10 showed strong anti-invasive activity	Yes

Abbreviations: ATC, anaplastic thyroid carcinoma; CYP24A1, cytochrome P450 family 24 subfamily A member 1; CYP27B1, cytochrome P450 family 27 subfamily B member 1 (1α-hydroxylase); DIO2, iodothyronine deiodinase type 2; EMT, epithelial–mesenchymal transition; FT3, free triiodothyronine; FT4, free thyroxine; HT, Hashimoto’s thyroiditis; IHC, immunohistochemistry; PTC, papillary thyroid carcinoma; RT-qPCR, quantitative reverse-transcription polymerase chain reaction; SMRT, silencing mediator of retinoid and thyroid hormone receptors; TSH, thyroid-stimulating hormone; VDR, vitamin D receptor; 25(OH)D, 25-hydroxyvitamin D; 1,25(OH)_2_D, 1,25-dihydroxyvitamin D. Table 2 focuses on experimental models, molecular pathways, biological outcomes, and limitations affecting translation to human thyroid disease.

## Data Availability

The original contributions presented in this study are included in the article. Further inquiries can be directed to the corresponding author(s).

## Abbreviations

The following abbreviations are used in this manuscript:

1,25(OH)2D3	1,25-dihydroxyvitamin D3
25(OH)D	25-hydroxyvitamin D
AI	Artificial intelligence
ALP	Alkaline phosphatase
ATD	Autoimmune thyroid disease
BMD	Bone mineral density
BMI	Body mass index
CYP	Cytochrome P450
CYP2R1	Cytochrome P450 family 2 subfamily R member 1
CYP24A1	Cytochrome P450 family 24 subfamily A member 1
CYP27B1	Cytochrome P450 family 27 subfamily B member 1
GC	Vitamin D-binding protein gene
HbA1c	Glycated hemoglobin
HT	Hashimoto’s thyroiditis
IFN-γ	Interferon-gamma
IGF-1	Insulin-like growth factor 1
IL-17	Interleukin-17
PTH	Parathyroid hormone
PR	Post-radioiodine
PRISMA	Preferred Reporting Items for Systematic Reviews and Meta-Analyses
PT	Post-thyroidectomy
RAI	Radioactive iodine
SMRT	Silencing mediator of retinoid and thyroid hormone receptors
SNPs	Single-nucleotide polymorphism(s)
T3	Triiodothyronine
T4	Thyroxine
TgAb	Thyroglobulin antibodies
Th1	T helper 1
Th17	T helper 17
TNF-α	Tumor necrosis factor-alpha
TPO	Thyroid peroxidase
TPOAb	Thyroid peroxidase antibodies
TRAb	Thyrotropin receptor antibodies
TSH	Thyroid-stimulating hormone
VDBP	Vitamin D-binding protein
VDR	Vitamin D receptor
VDREs	Vitamin-D-responsive Regulatory Elements
